# Effective editing for lysophosphatidic acid acyltransferase 2/5 in allotetraploid rapeseed (*Brassica napus* L.) using CRISPR-Cas9 system

**DOI:** 10.1186/s13068-019-1567-8

**Published:** 2019-09-20

**Authors:** Kai Zhang, Liluo Nie, Qiqi Cheng, Yongtai Yin, Kang Chen, Fuyu Qi, Dashan Zou, Haohao Liu, Weiguo Zhao, Baoshan Wang, Maoteng Li

**Affiliations:** 1 Department of Biotechnology, College of Life Science and Technology, Huazhong University of Science and Technology, Wuhan, China; 2 Hubei Key Laboratory of Economic Forest Germplasm Improvement and Resources Comprehensive Utilization, Hubei Collaborative Innovation Center for the Characteristic Resources Exploitation of Dabie Mountains, Huanggang Normal University, Huanggang, China; 3 College of Life Science, Shandong Normal University, Jinan, 250000 China

**Keywords:** CRISPR-Cas9, *BnLPAT2*, *BnLPAT5*, Seed oil, Allotetraploid, *Brassica napus*

## Abstract

**Background:**

*Brassica napus* is one of the most important oilseed crops, and can supply considerable amounts of edible oil as well as provide raw materials for the production of biodiesel in the biotechnology industry. Lysophosphatidic acid acyltransferase (LPAT), a key enzyme in the Kennedy pathway, catalyses fatty acid chains into 3-phosphoglycerate and promotes further production of oil in the form of triacylglycerol. However, because *B. napus* is an allotetraploid with two subgenomes, the precise genes which involved in oil production remain unclear due to the intractability of efficiently knocking out all copies with high genetic redundancy. Therefore, a robust gene editing technology is necessary for gene function analysis.

**Results:**

An efficient gene editing technology was developed for the allotetraploid plant *B. napus* using the CRISPR-Cas9 system. Previous studies showed poor results in either on-target or off-target activity in *B. napus*. In the present study, four single-gRNAs and two multi-gRNAs were deliberately designed from the conserved coding regions of *BnLPAT2* which has seven homologous genes, and *BnLPAT5*, which has four homologous genes. The mutation frequency was found to range from 17 to 68%, while no mutation was observed in the putative off-target sites. The seeds of the *Bnlpat*2/*Bnlpat*5 mutant were wizened and showed enlarged oil bodies, disrupted distribution of protein bodies and increased accumulation of starch in mature seeds. The oil content decreased, with an average decrease of 32% for *Bnlpat2* lines and 29% for *Bnlpat5* lines in single-gRNA knockout lines, and a decline of 24% for *Bnlpat2* mutant lines (i.e., g123) and 39% for *Bnlpat2*/*Bnlpat5* double mutant lines (i.e., g134) in multi-gRNA knockout lines.

**Conclusions:**

Seven *BnLPAT2* homologous genes and four *BnLPAT5* homologous genes were cleaved completely using the CRISPR-Cas9 system, which indicated that it is effective for editing all homologous genes in allotetraploid rapeseed, despite the relatively low sequence identities of both gene families. The size of the oil bodies increased significantly while the oil content decreased, confirming that *BnLPAT2* and *BnLPAT5* play a role in oil biosynthesis. The present study lays a foundation for further oil production improvement in oilseed crop species.

## Background

Triacylglycerol (TAG) is the main storage oil in seeds and also a good resource for biofuels, lubricants and industrial feedstocks [1]. The production of plant oil is a necessity to meet the increasing global demand for edible oil and biofuel. *Brassica napus*, as one of the most important edible oilseed crops, has gained significant attention worldwide for its considerable amounts of edible oil for nutrition. It is not only rich in oleic acid contents (78–88% of the oil), but also high in oil contents (40–45% of the mass) [2, 3]. Therefore, optimizing the composition and maximizing the yield of oils in crops are of great importance for plant breeders and the biotechnology industry.

The fatty acyl-CoA-dependent Kennedy pathway, which guides three fatty acyl moieties to attach to a glycerol backbone, is clearly understood [4, 5]. Glycerol-3-phosphate acyltransferase (GPAT), lysophosphatidic acid acyltransferase (LPAT) and diacylglycerol acyltransferase (DGAT) are involved in TAG biosynthesis in that order [4]. Notably, LPAT is in a crucial position not only for the biosynthesis of TAG by dephosphorylation, but also for the formation of membrane lipids [4]. Based on previous QTL mapping results for oil content in our group, *BnLPAT2* and *BnLPAT5* have been identified as potential genes located in candidate regions of three natural populations (that is, the KN and TN population) of *B. napus* [6–8]. Therefore, it is necessary to study the functions of *BnLPAT2* and *BnLPAT5* in *B. napus*. Kim et al. studied the four cytoplasmic *AtLPAT* (LPAT2-5) genes of *Arabidopsis thaliana*, and found that heterozygous mutants of *LPAT2* would produce shorter siliques as well as cause abortion in the female gametophyte [9]. Chen et al. found that overexpression of *RcLPAT2* would increase the accumulation of ricinoleic acid (18:1OH) at the sn-2 position of LPA in the transgenic *Lesquerella* seeds [10]. Overexpression of *AtLPAT1*-*5* under phosphate starvation revealed that only *AtLPAT2* could significantly contribute to root development, and a significantly increased level of PC and PE in rosette leaves in *Arabidopsis* [11]. To date, the function of LPATs has not been reported in *B. napus* or its diploid progenitors, *B. oleracea* and *B. rapa*.

*Brassica napus* (2*n* = 38, AACC) is an allotetraploid species that originated from a hybridization between *B. rapa* (2*n* = 20, AA) and *B. oleracea* (2*n* = 18, CC) [12]. Consequently, no obvious effects could be achieved by overexpressing its genes due to their intrinsic high expression from multiple copies in *B. napus* compared to the diploid *Arabidopsis*. Additionally, the modification of particular gene is limited by their potential redundant functions [13]. Therefore, it is important to knock out all the homologous genes from both the A and C subgenomes to obtain a reliable genotype or phenotype in rapeseed [14] and other polyploid plants [15, 16]. Genome editing technology has allowed more attention in the last few years in polyploid species. Three cleavage systems (protein-dependent cleavage system, DNA-dependent cleavage system and RNA-dependent cleavage system) have been widely used in genome editing [17–19]. Both zinc-finger nucleases (ZFNs) and transcription activator-like effector nucleases (TALENs) have been successfully applied in many plants, such as *A. thaliana*, rice, wheat, soybean and maize, but they have not been successfully applied in *Brassica* species [20–24]. It is complex to construct target vectors suitable for protein-to-DNA recognition. The advent of the inexpensive, efficient and versatile CRISPR-Cas9 system has attracted great interest in multi-copy gene knockout in recent years. However, the complete knock out of all copies of one functional gene has remained intractable due to high genetic redundancy [25]. At present, the simultaneous multi-copy knockout of a target gene by CRISPR-Cas9-mediated cleavage has been shown in some polyploid plant species, such as hexaploid bread wheat, cotton, switchgrass and *Camelina sativa* [15, 26–28].

Although efforts have been made towards CRISPR-Cas9-mediated cleavage in *Brassica* species, it is far from gene modification to comprehensive application in crop improvement. For example, Lawrenson et al. designed an sgRNA to targeted *BolC.GA4.a* which has two nucleotide mismatches in its paralogous gene *BolC.GA4.b*, and found that 10% (2/20) *ga4* mutants were obtained, while 36% (32/68) of the T1 progeny revealed off-target activity in *BolC.GA4.b* with CRISPR-Cas9-mediated mutations in *B. oleracea* [29]. Braatz et al. designed a gRNA for only the A homologue of *BnALC*, which resulted in a cleavage of both the A and C homologues in *BnALC* at only one obtained T0 mutant plantlet in *B. napus* [25]. It is obvious that gene editing in both the A homologue (that is, on-target effect) and C homologue (that is, off-target effect) occurred simultaneously, which is not conducive to efficient genome editing. In addition, Yang et al. reported that sgRNA1 could result in 14.4% editing efficiency in both the A and C homologues, despite one mismatch in the target site in *B. napus* [30]. However, all these results were established in a limited number of obtained mutant plantlets or with lower mutant efficiency which might make these methods unreliable for intensive study. Currently, the majority of studies have focused on multi-locus targets with multi-gRNA in the CRISPR-Cas9 system. There have not been intensive studies that focus on multi-copy knockout with CRISPR-Cas9 in *B. napus*. In addition, some research have revealed that two gRNAs in one construct for single-gene targeting yield a good result, while four gRNAs in one construct could not yield a reliable result [30, 31]. The detailed relationship between the gRNA and the gene loci has not been elucidated in allotetraploid *B. napus*.

In the present study, four single-gRNA and two multi-gRNA constructs were designed for *BnLPAT2* and *BnLPAT5*, which share generalized sequence similarity and participate in the oil synthesis pathway. All homologous genes of *BnLPAT2* and *BnLPAT5* were completely knocked out without off-target effects using CRISPR-Cas9-mediated gene editing techniques. Simultaneous knock out of at least four copies of target genes in both the A and C subgenomes, which did not share extremely high similarity, has not reported before in *B. napus*. In addition, insertions, deletions, substitutions and combined mutation types in the target loci were obtained from 736 target samples. Importantly, no editing occurred at the putative off-target sites. Consequently, the oil content decreased by 24–39%, and obvious changes in FA composition as well as enlarged oil bodies were observed in the present study. The present study offers a better understanding of accurate target mutation and gene function as well as gene identification in rapeseed and other polyploid species.

## Results

### Comparison and characterization of *Brassica napus* LPAT genes

**Fig. 1:**
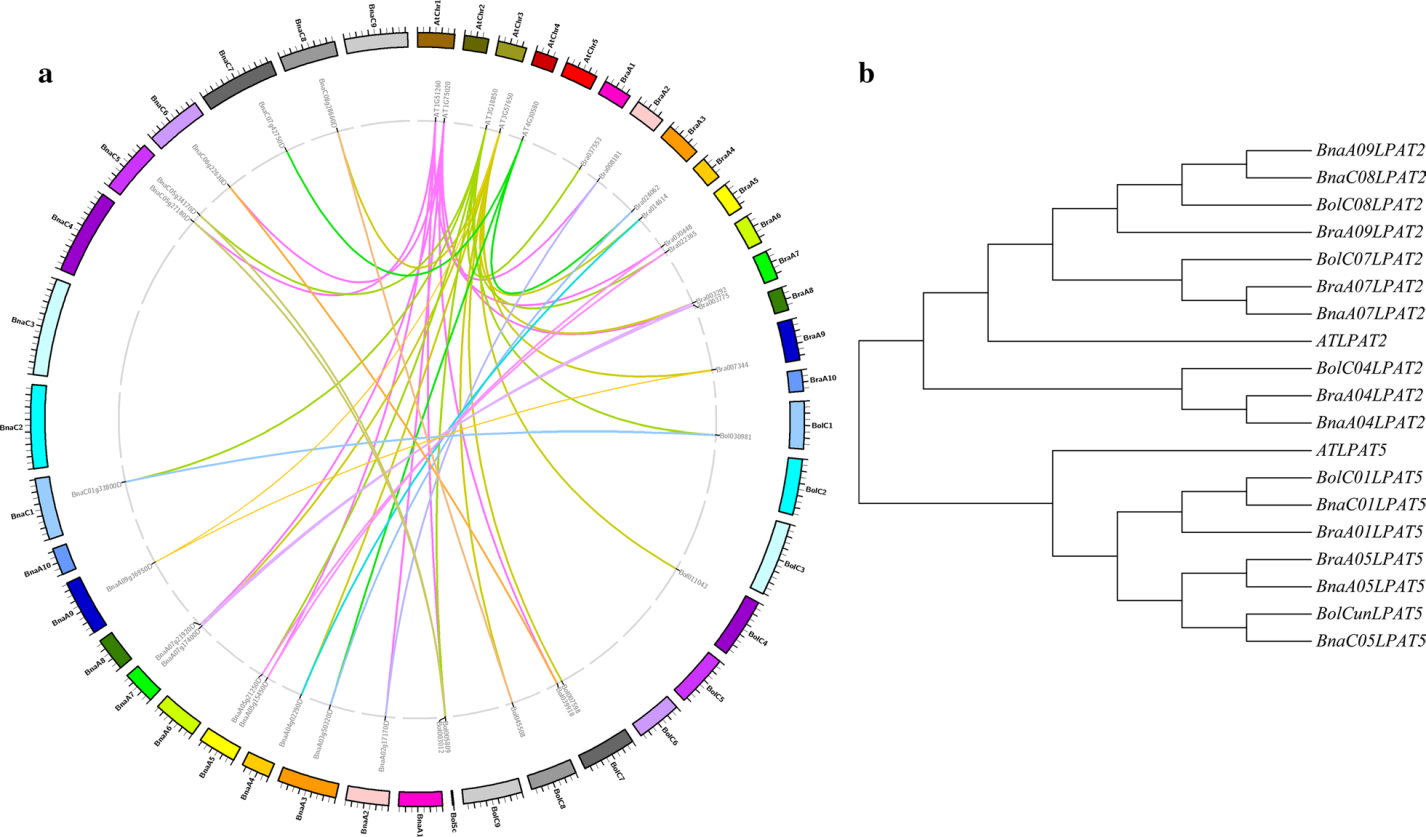
Synteny analyses and phylogenetic relationships of LPAT homologous genes in four related Brassicaceae species. **a** Synteny analyses of LPAT homologous genes from *B. napus* and other related species. **b** Phylogenetic tree of LPAT2 and LPAT5 homologous genes from *B. napus* and other related species

To study the changes in LPAT genes during the genome multiplication process, synteny and phylogenetic relationships of all *LPAT* homologous genes from four related Brassicaceae species (*A. thaliana*, *B. oleracea*, *B. rapa* and *B. napus*) were studied. Their protein sequences were retrieved from the Brassica database (http://brassicadb.org/brad/searchSyntenytPCK.php). The synteny analysis revealed that the *BnLPAT* genes of *B. napus* are closely related to other *LAPT* genes in *A. thaliana*, *B. oleracea* and *B. rapa*. Additionally, 1, 3, 3 and 4 homologous genes of *LPAT2*, 1, 2, 2 and 3 homologous genes of *LPAT5* were obtained in the four related Brassicaceae species above, respectively (Fig. 1a, b). Further analysis revealed that the *BnLPAT2* s were located in chrA09, chrA04, chrA07 and chrC08, while the *BnLPAT5* s were located in chrA05, chrC05 and chrC01 in *B. napus* (Fig. 1b). According to the phylogenetic analysis for LPAT2/LPAT5, all species except for *Arabidopsis* had at least two LPAT orthologues (Fig. 1). LPAT gene duplication or triplication events might have occurred in these species, which implies that LPAT has followed a complex evolutionary trajectory. Thus, we speculated that *BnLPAT2* and *BnLPAT5* are of great importance for TAG accumulation in the Kennedy pathway.

Next, to further study the gene sequences of *BnLPAT2* and *BnLPAT5* in *B. napus*, seven and four homologous genes of *BnLPAT2* and *BnLPAT5* were obtained from the *B. napus* genome database (http://www.genoscope.cns.fr/brassicanapus/) according to their sequence identities, respectively. The sequence alignments revealed that both the *BnLPAT2* and *BnLPAT5* gene families had high homology with their counterparts (Additional files 1, 2). Relative to *Bna*A4*LPAT*2, the *Bn*aA7*LPAT*2, *Bn*aA9*LPAT*2, *Bna*C4*LPAT2*, *Bna*C7*LPAT*2, *Bna*C8*LPAT*2 and *Bna*A07*LPAT*2 genes have sequence identities of 89.8%, 90.6%, 83.5%, 89.8%, 90.5% and 70.5%, respectively (Additional file 3). Compared to *Bna*A5*LPAT*5, the sequence identities of *Bna*C1*LPAT*5, *Bna*C5*LPAT*5 and *Bna*UK*LPAT*5 were 84.8%, 97.4% and 91.3%, respectively (Additional file 3). Furthermore, multiple sequence alignment using the DNAMAN software showed that the identities of the *BnLPAT2* and *BnLPAT5* families were 60.0% and 77.8%, respectively. The relatively low identity between *BnLPAT2* and *BnLPAT5* indicated that they might possess different functions in TAG accumulation.

### CRISPR-Cas9 via high-efficiency *Agrobacterium*-mediated hypocotyl transforming system in *B. napus*

**Fig. 2:**
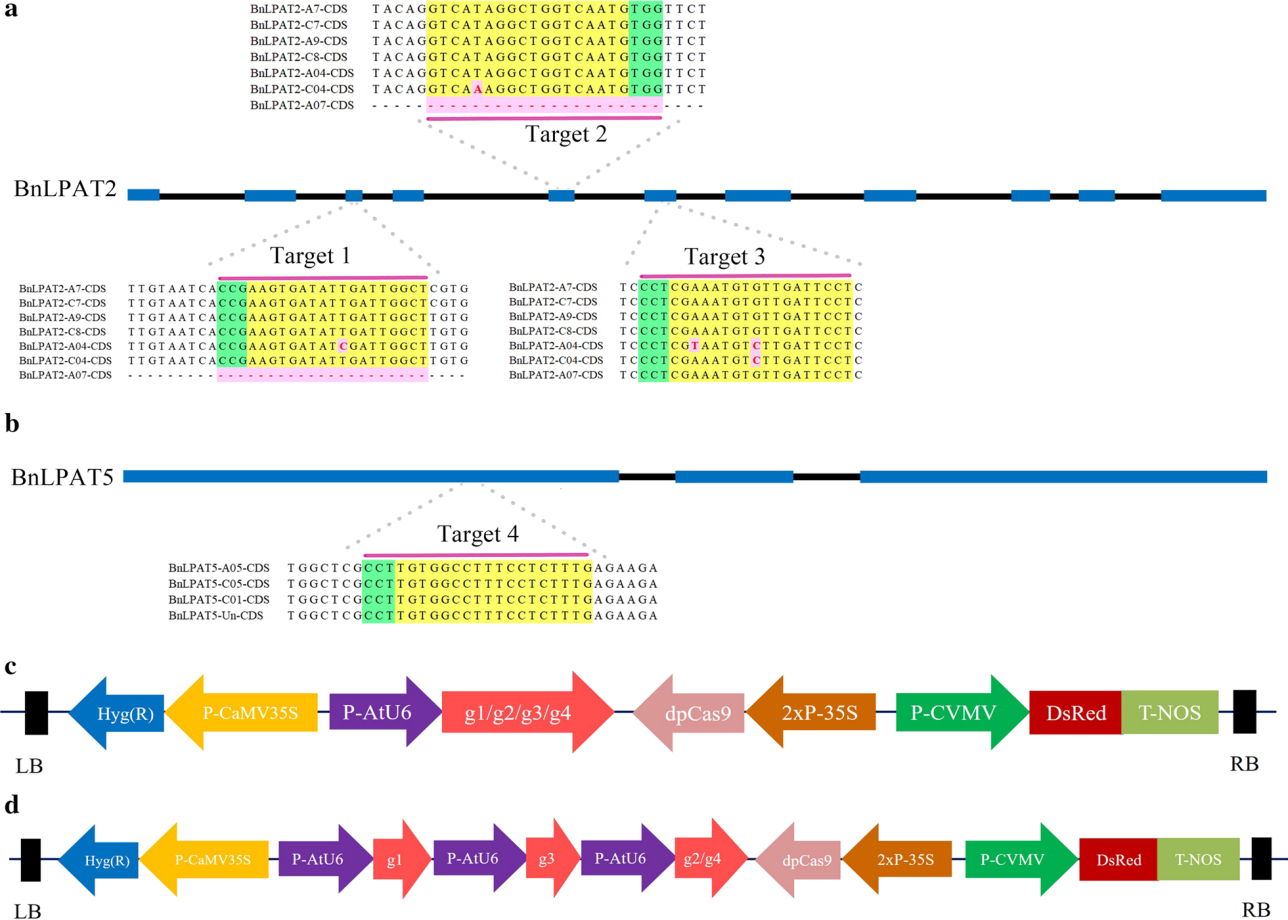
*BnLPAT* gene models with target sequences and schematic of binary vectors. **a**, **b** Diagram of the *BnLPAT2* and *BnLPAT5* gene models. Exons are shown with blue rectangles, whereas introns are shown with black lines. Target sites in the conserved CDS region of *BnLPAT* genes. SNPs are highlighted in light red. PAMs are coloured green, whereas gRNAs are coloured yellow. **c**, **d** Diagram of the single-gRNA- and multi-gRNA-mediated CRISPR-Cas9 constructs. The expression of the Cas9 protein is driven by the 2× 35S promoter with a 2× nuclear localization signal (NLS) at the N terminus. The *Arabidopsis* promoter U6-26 is used to drive the sgRNA expression

To knock out all the homologous genes of *BnLPAT2* and *BnLPAT5* simultaneously, four target sites were deliberately selected in the conserved CDS region (Fig. 2a, b). Target 1, target 2 and target 3 were located in exons 3, 5 and 6 of *BnLPAT*2, respectively, whereas target 4 was located in exon 1 of *BnLPAT*5. These target sequences were oriented in the 5′ to 3′ direction of both the forward strand and reverse strand, while the protospacer adjacent motif (PAM) was located in either the 5′ or 3′ region of the target sequences. Each site was deliberately selected to locate in front of 50% of each sequence to ensure that the open reading frames resulting from disruptions of the interest gene would result in products lacking enzymatic activity. Notably, each target covered five copies, whereas target 1 and target 2 contained an SNP located 10 bp upstream of the PAM in *Bna*A4*LPAT*2 and 14 bp upstream of the PAM in *Bna*C4*LPAT*2, respectively. In target 3, however, the SNPs were 9 bp upstream of the PAM in *Bna*C4*LPAT*2 and 3 bp and 9 bp upstream of the PAM in *Bna*A4*LPAT*2.

Four 18 nt oligos (21 nt including the NGG PAM region) that targeted *BnLPAT2* or *BnLPAT5* were separately cloned into the CRISPR-Cas9 construct. Then, four single-gRNA binary constructs, *Bn*LPAT2-target1 (hereafter, named g1), *Bn*LPAT2-target2 (hereafter, named g2), *Bn*LPAT2-target3 (hereafter, named g3) and *Bn*LPAT5-target4 (hereafter, named g4) were designed to target *BnLPAT2* and *BnLPAT5*, respectively (Fig. 2c). An in vitro assay showed that the Cas9 nucleosidase activities of g1, g2, g3 and g4 were 77%, 38%, 64% and 63%, respectively (Additional file 4), which indicated that the Cas9 nuclease was active in target sequence cleavage. In addition, to more effectively obtain mutated transgenic lines, two multi-gRNA binary constructs, g1–g2–g3 (hereafter, named g123) and g1–g3–g4 (hereafter, named g134), were designed for multiple gene knockout (Fig. 2d). In each construct, the gRNA scaffold was under the control of the *Arabidopsis* Ubiquitin 6-26 promoter (*At*U6-26), while the Cas9 scaffold was under the control of the *Cauliflower mosaic* virus 35S (CaMV35S) promoter. In addition, all these constructs were independently transformed into hypocotyl tissue to generate callus at the resistance of hygromycin for rapid selection.

After transformation into the semi-winter cultivar of J2016 using the *Agrobacterium*-mediated hypocotyl transformation method, 247 complete plantlets were obtained through callus formation, bud differentiation and rooting culture. In detail, 25, 48, 41, 93 30 and 10 independent lines for g1, g2, g3, g4, g123 and g134 survived as rooted plantlets. PCR amplification was performed using the gRNA scaffold primers, gRNA-F and gRNA-R to identify transgene-positive T0 plants (Additional files 5, 6), and it was shown that 84% (21/25) of g1, 81% (39/48) of g2, 98% (40/41) of g3, 97% (87/93) of g4, 100% (30/30) of g123 and 100% (10/10) of the g134 T0 plantlets carried gRNA scaffold integration (Table 1). The average transgenic efficiency was 93%. Furthermore, Sanger sequencing was performed to validate the mutation rate, and the double peaks in the sequencing chromatogram at the target site were observed. Specifically, 68% (59/93) mutation efficiency was observed in g4, followed by g2 (41%, 16/48), g134 (30%, 3/10), g1 (29%, 6/25), g3 (28%, 11/41) and g123 (17%, 5/30) (Table 1). It was also found that the 50% GC content of g2 and g4 showed higher mutation efficiency than the 33% and 39% of g1 and g3, respectively. The efficiency of transgene insertion and mutation demonstrated that an effective *Agrobacterium*-mediated hypocotyl transformation system was beneficial to CRISPR-Cas9-mediated knock-out in *B. napus*.

**Table 1:** Transformation efficiencies of different gRNAs in CRISPR-Cas9-mediated transgenic lines

	gRNA-sc	Mutant	Total	Transgenic efficiency (%)	On-target efficiency (%)
g1	21	6	25	84	29
g2	39	16	48	81	41
g3	40	11	41	98	28
g4	87	59	93	97	68
g123	30	5	30	100	17
g134	10	3	10	100	30

### Single-gRNA-mediated mutation using CRISPR-Cas9 in *B. napus*

**Fig. 3:**
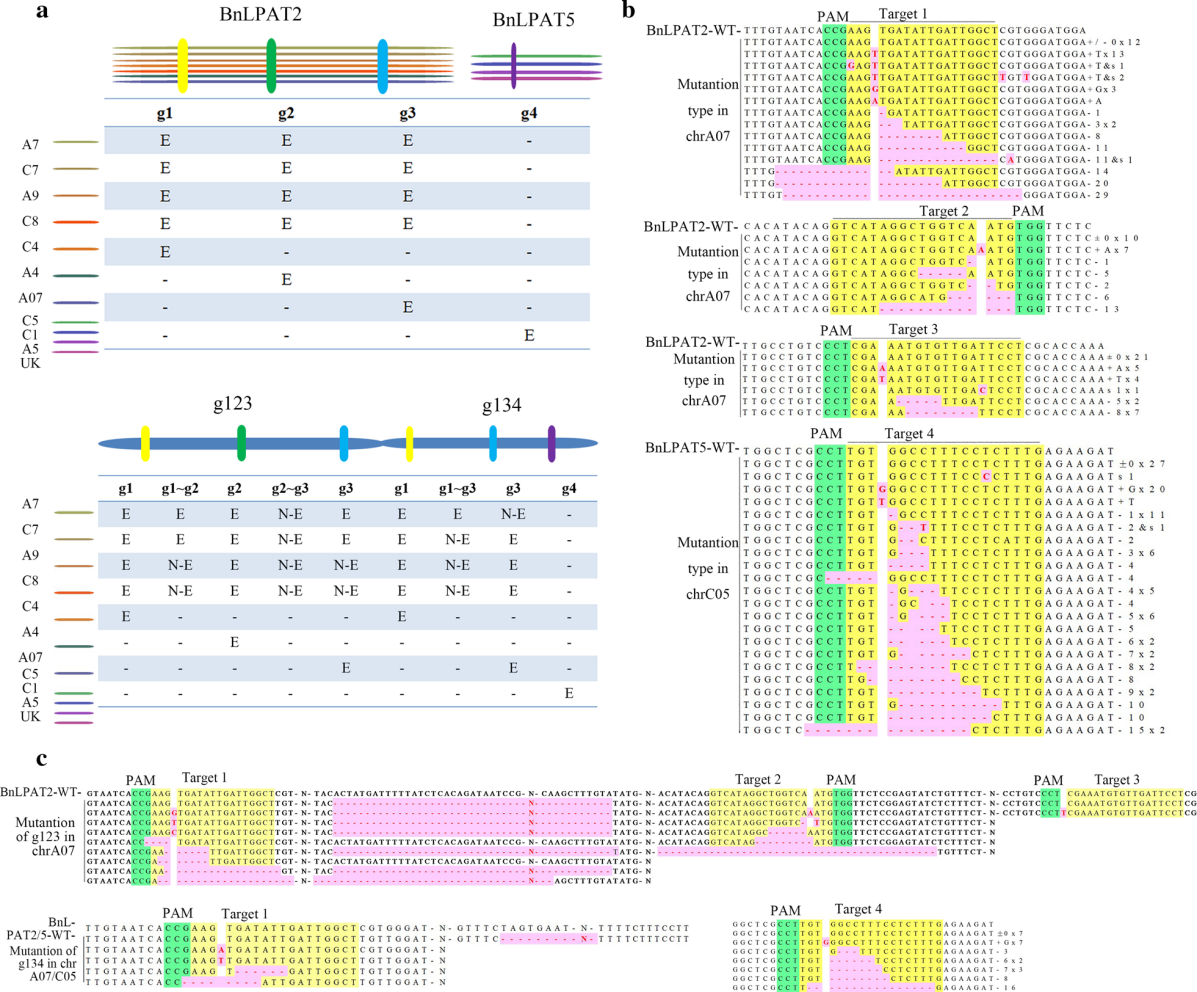
Schematic of gene editing using CRISPR-Cas9 in *B. napus*. **a** Single-gRNA (upper) and multi-gRNA (lower) mediated gene editing in *B. napus*. E, editing; N-E, non-editing. **b** Single-gRNA mediated target for knockout at CRISPR-Cas9-mediated cleavage. Sequencing results in chrA07 are listed in g1 lines, g2 lines and g3 lines, respectively, and in the common site of g4 lines. **c** Multi-gRNA-mediated target for knockout at CRISPR-Cas9-mediated cleavage. “−” and “+” indicate nucleotide insertions and deletions in the target sequence, while “s” indicates substituted mutations, and “c” indicates combined mutations. The pentagram in red indicates insertions in the target site. gRNAs are coloured yellow, whereas PAMs are coloured green

To characterize the mutations of the target sequence in detail, 4, 4, 5 and 10 mutated plantlets of g1, g2, g3 and g4 were selected for further sequencing. The sequences contained target sites from seven homologous *BnLPAT2* genes, and the conserved regions of four homologous *Bn*LPAT5 genes were amplified and then cloned into the pMD18-T vector. As a result, 65 vectors for *BnLPAT2* and 10 for *BnLPAT5* were constructed. After cocultivation transformation, eight to ten positive single colonies on each plate were picked randomly for sequencing. As expected, indels were detected in all the homologous genes in the target region, which indicated that the CRISPR-Cas9 system is highly efficient in knocking out all homologous genes in allotetraploid *B. napus* (Fig. 3a, b and Additional file 7).

**Fig. 4:**
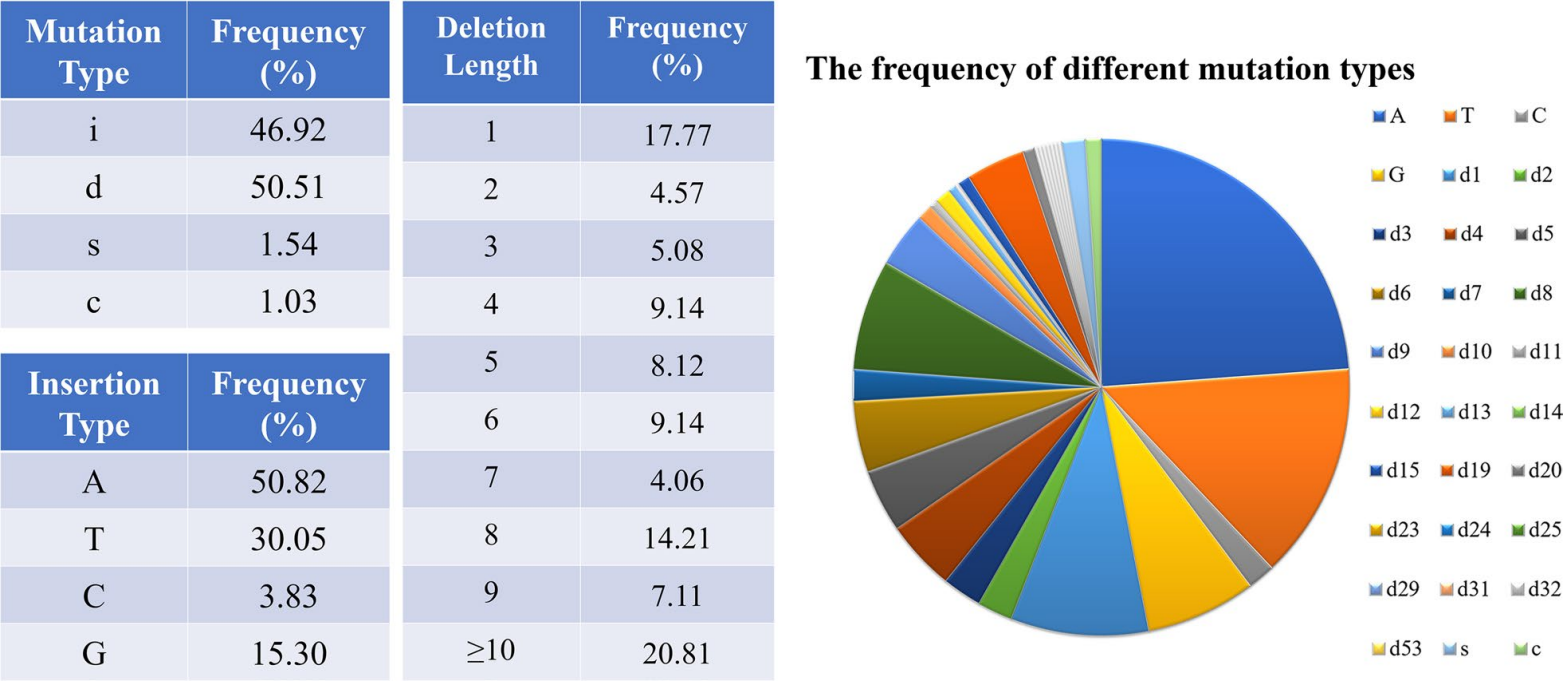
Detailed analysis of single-gRNA-mediated mutations in T0 plants. Different mutation types are shown in the left table, while the frequency of different insertion types is shown in the middle table and the frequency of different deletion types are shown in the right table. The pie graph indicates different mutation types of all single-gRNA-mediated mutations

Among the 612 sequenced samples obtained, 390 were mutated at the target site. Four mutation types were obtained: 47% deletion, 51% insertion, 2% substitution and 1% combined mutation (Fig. 4). Further analysis revealed that all the insertions were 1 bp insertion, with a distinct preference for A (51%) over other nucleotides. They all occurred at 3 bp proximal to the PAM at the target sites. The deletions varied from − 1 to − 53 bp, and the short deletions had a higher frequency than the long deletions (Fig. 4). Notably, almost all the − 1 bp deletions occurred at 3 bp proximal to the PAM, whereas the others were random and even crossed the PAM region (Fig. 3b and Additional file 7). Substitutions such as T → C, T → A, and A → G occurred irregularly at the target site (Fig. 3b and Additional file 7). To a certain extent, T → C conversions were more favoured than T → A (only in g1-chrC7) or A → G (only in g3-chrA9) conversions, because T → C conversions was in the frequency of 67% that occurred in three of the four targets (i.e., in g1-chrC8/C4, g3-chrA7 and g4) (Fig. 3b and Additional file 7). These conversions indicated that the substitutions induced by CRISPR-Cas9 could be equal to those of the cytidine deaminase recognized as a “base editor” [32, 33]. The frequency of combined mutations was 1%, which suggested nonuniform cell division during meiosis. However, several mutated genotypes co-existed in one T0 plant, indicating that chimaerism occurred at a higher frequency. For example, in *Bna*A7*LPAT2*, thirteen, six and five mutation types were obtained for g1, g2 and g3, respectively (Fig. 3b). This result indicated that more than one mutation occurred in a target site. Consequently, all copies of the target gene from both A and C homoeologues were knocked out with this CRISPR-Cas9 system even though they possessed multiple sequences.

### Multiple-gRNA-mediated mutation using CRISPR-Cas9 in *B. napus*

Knocking out all the homologous genes of a gene family simultaneously is of great importance for phenotype modification, especially in allotetraploid plants. Thus, four mutated multi-gRNA lines of g123 and three mutated lines of g134 were selected for further sequencing. All the homologous genes containing the three target sites were independently amplified for *BnLPAT2*, while the conserved region of four homologous *BnLPAT5* genes was amplified for TA cloning. As a result, 206 samples for g123 and 140 samples for g134 were obtained. In multi-gRNA-mediated cleavage, all the homologous genes were edited at the target sites (Fig. 3a, c and Additional file 8). The mutation types were consistent with single-gRNA-mediated mutations. In addition, there were large fragment deletions between g1 and g2 as well as g2 and g3 in *BnLPAT2*. Notably, mutations in target 3 of *BnLPAT2* occurred at single-gRNA g3 and multiple-gRNA g134, but not at g123 (Fig. 3a and Additional file 8). However, the difference of target 3 between g123 and g134 was that the former was in the third site, whereas the latter was in the second site of *BnLPAT2* gene. This result might indicate that target 3 was active for cleavage only when it was located before the first three sites of one target sequence. Therefore, the efficacy of CRISPR-Cas9 would be limited by the number of target sites in one gene. Overall, two genes were knocked out by the CRISPR-Cas9 system, which might offer great prospects for multiple gene mutations that share relatively low sequence identity.

### Off-target activity in CRISPR-Cas9-mediated mutation in *B. napus*

To explore the off-target mutagenesis occurring with the CRISPR-Cas9-induced mutation, the genome-wide potential off-target sites were searched using the CRISPR RGEN Tools website (http://www.rgenome.net/cas-offinder/) for all target sites, and then mapped to the *B. napus* genome (http://www.genoscope.cns.fr/brassicanapus/) to obtain the matching gene sequence. Several potential off-target sites with no more than three SNPs’ difference from the target site were obtained in *B. napus* (Table 2). Among all the potential off-target sites searched, g1-OFF1 and g3-OFF1 exactly matched the site in *Bna*A4*LPAT2*, whereas g2-OFF2 and g3-OFF5 matched *Bna*C4*LPAT2*, indicating that the off-target sites were reliable. All these sites were located in the exon region according to the Darmor-bzh reference genome. Then, PCR primers were designed for amplifying fragments covering potential off-target sites (Additional file 6). Consequently, the PCR products of these off-target sites from the mutated plant above were sequenced, and the results were then aligned to the Darmor-bzh reference genome to search for SNP sites. As expected, the off-target sites corresponding to one to two mismatches were detected with no genome editing (Table 2). These results indicated that this CRISPR-Cas9 system performed high-quality genome editing accompanied by undetectable off-target activity in *B. napus*.

**Table 2:**
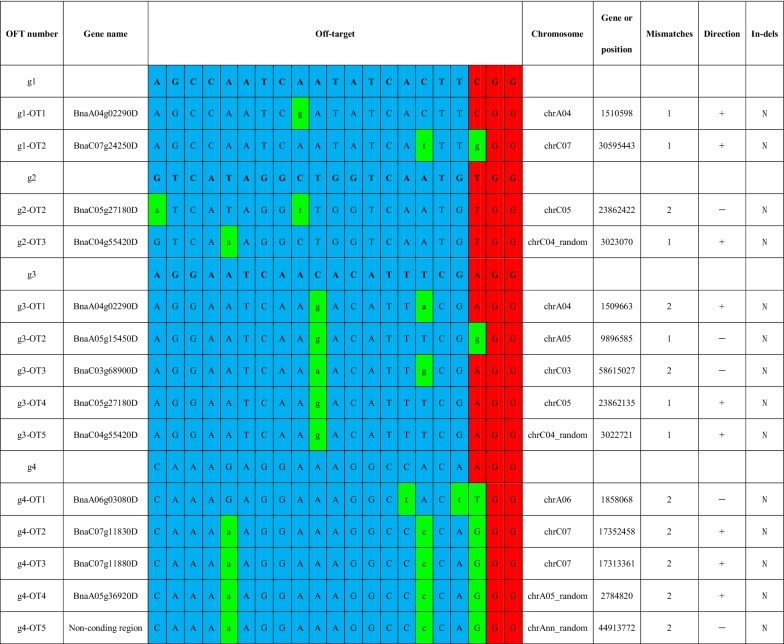
Potential off-target activity for each sgRNA target site in transgenic *B. napus*

Nucleotides in the green background are consistent with the designed sgRNA target sites. Mismatched nucleotides are in lower case. The PAM is highlighted with a red background

### Increased size of oil bodies in *Bnlpat2*/*Bnlpat5* mutant mature seed of *B. napus*

**Fig. 5:**
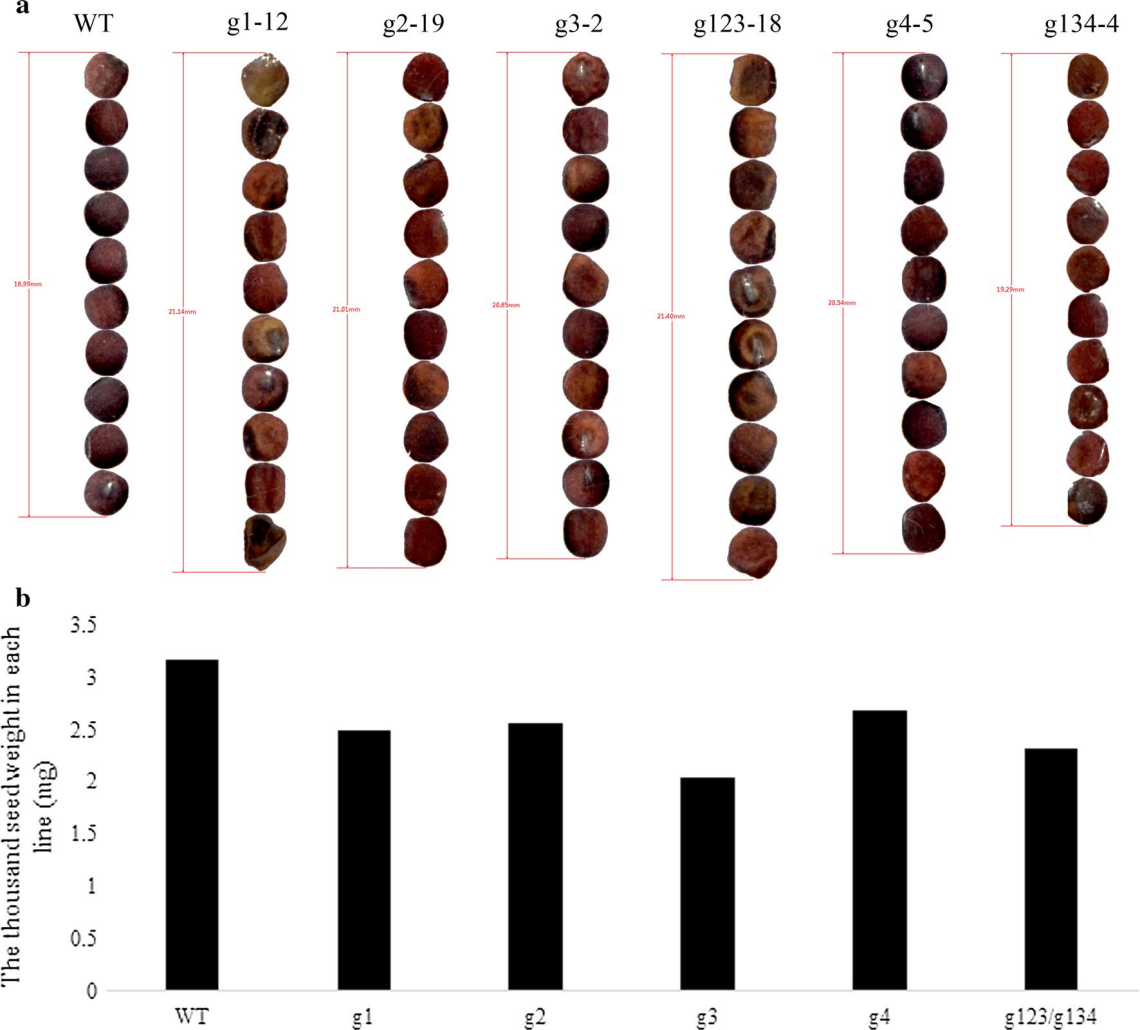
Seed morphology in dry mature seeds of *Bnlpat2*/*5* knockout lines. **a** Scan of seed morphology from largest to smallest according to the area. **b** Thousand seed weights in each mutant line

**Fig. 6:**
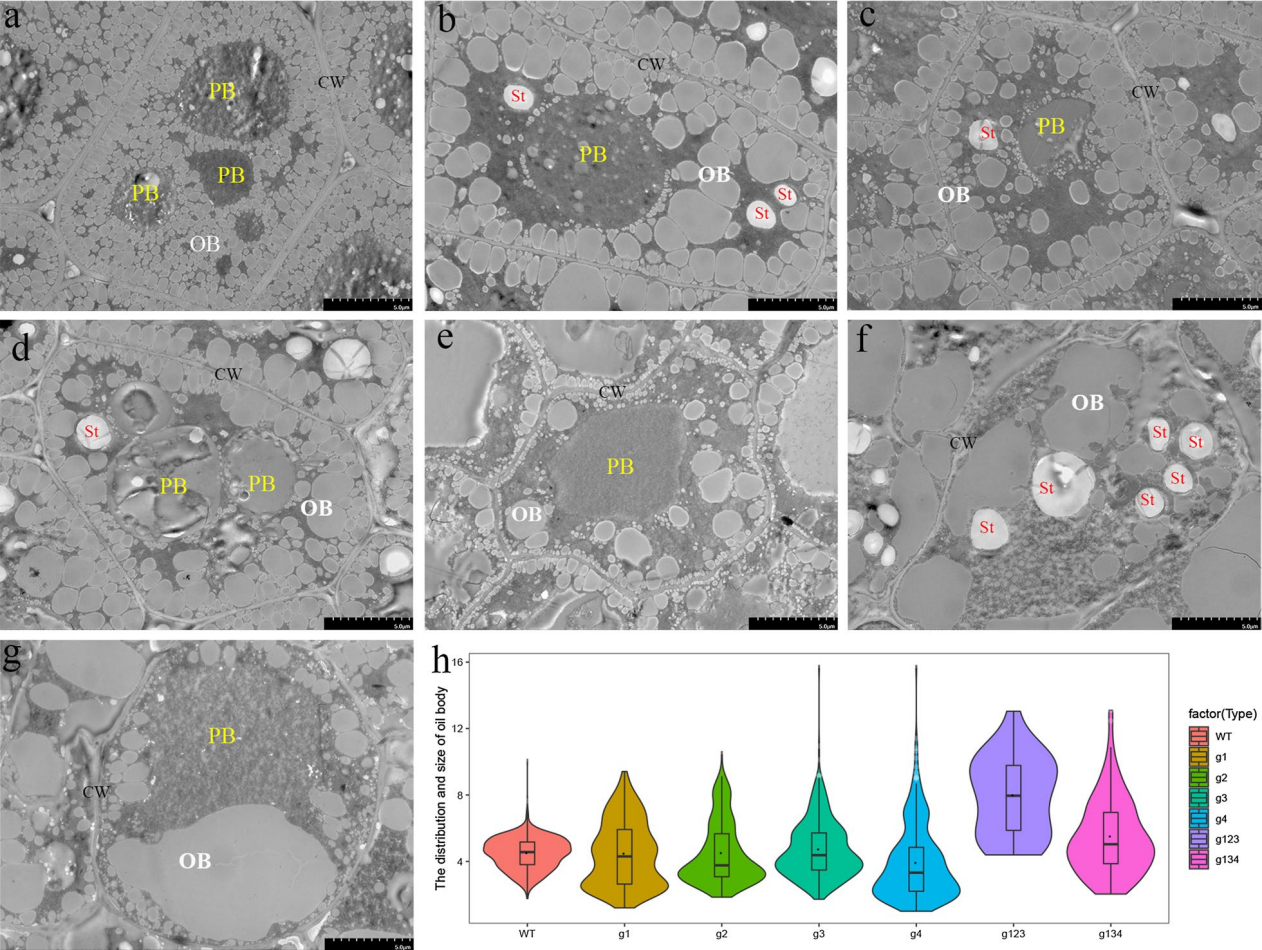
Ultrastructural study of *Bnlpat2*/*5* knockout lines in mature cotyledons. **a–g** represent the mutant lines in WT, g1, g2, g3, g4, g123 and g134. OB indicates oil body, PB indicates protein body, St indicates starch, CW indicates well wall. **h** represents the distribution and the size of oil body size

In the *Bnlpat2*/*Bnlpat5* mutant, the seed morphology was wizened to different degrees compared to WT (Fig. 5a), and the average thousand seed weights of g1, g2, g3, g4, g123 and g134 were decreased slightly compared to that of WT (Fig. 5b). To investigate the effects of *BnLPAT2* and *BnLPAT5* gene knockout on oil accumulation, mature seed microstructures of the *Bnlpat2/Bnlpat5* mutant were obtained by transmission electron microscopy (TEM). The oil bodies in the cotyledons of wild-type mature seeds were mostly uniform in size and present in the periphery of the cells or between protein bodies (Fig. 6a). However, differences were observed in *Bnlpat2*/*Bnlpat5* mutant seeds compared to wild-type seeds. Specifically, the oil bodies in *Bnlpat2*/*Bnlpat5* mutant seeds were heterogeneous in size, and the sizes of some oil bodies were increased greatly (Fig. 6b–g). According to the results processed by Image J software, the average size of the oil bodies in the g1, g2, g3 and g4 lines was twice than that of WT. Unusually, large oil bodies distributed in the periphery of the cells were also observed, especially in the g134 and g123 lines, some of which were 15 times and 30 times larger than that of wide type (Fig. 6f, g, Additional file 9). The present results showed that the change was much larger in multi-gRNA knockout lines than in single-gRNA knockout lines. In addition to these unusually large oil bodies, many unusually small oil bodies were also present in the g1, g2, g3 and g4 lines (Fig. 6b–e). Consequently, the distributions of oil bodies were highly irregular in mutant lines (Fig. 6h). Meanwhile, the total number of oil bodies also decreased, and further analysis revealed that the area ratio of all the oil bodies in the cell also decreased, which was 0.26–0.40 times lower in the mutant lines than in the WT (Additional file 9). In addition to the oil body, the organization of protein bodies was also affected, and it was variously irregular in the centres of cells. In addition, starch also accumulated in the mature seeds of *Bnlpat2/Bnlpat5* knockout lines, and the cell wall also became irregular and thinner than that of WT. Collectively, the present results showed that the knockout of *Bnlpat2/Bnlpat5* could result in a perturbation of oil body biogenesis.

### Decreased oil content in *Bnlpat2*/*Bnlpat5* mutant mature seeds of *B. napus*

**Fig. 7:**
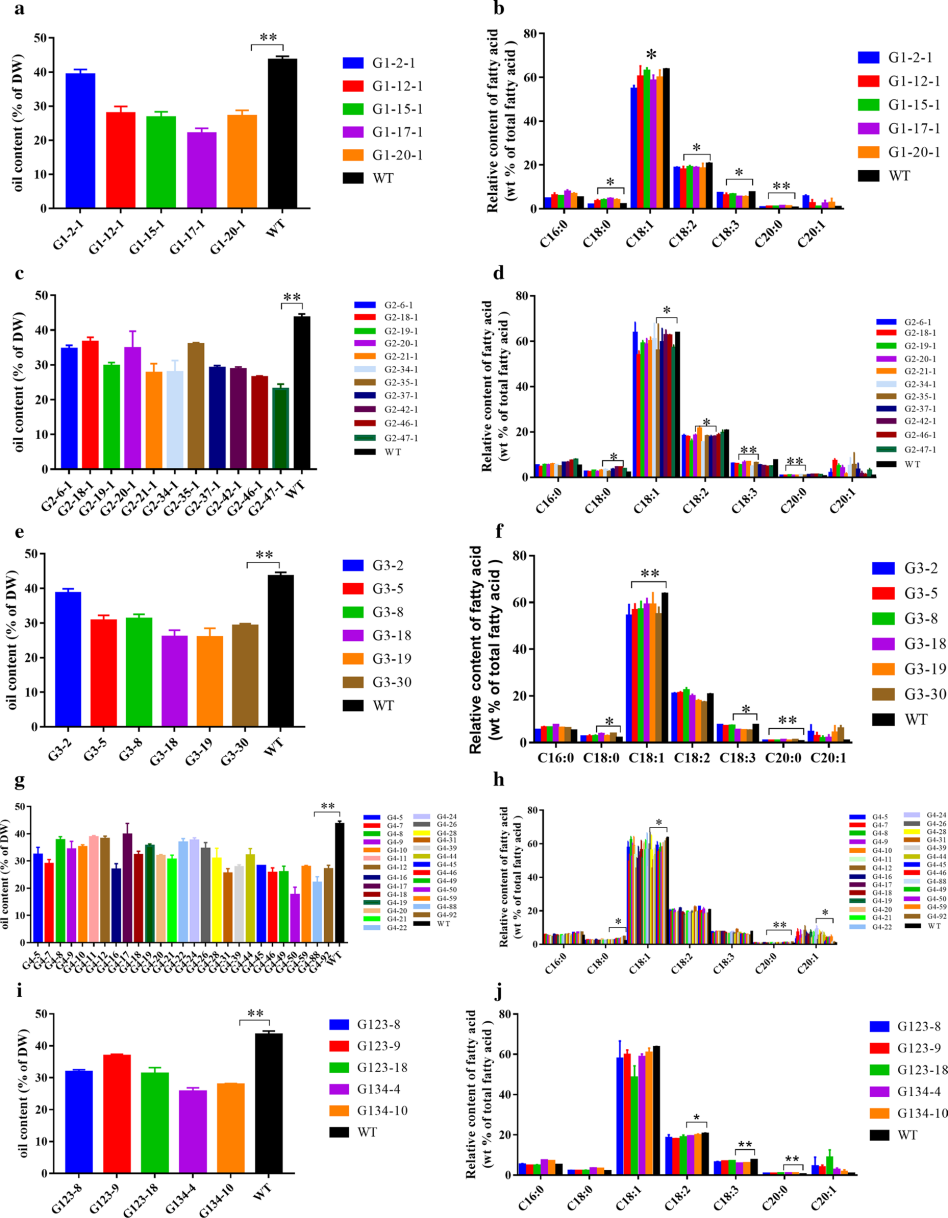
Oil content and relative fatty acid composition in knock-out mutant of *Bnlpat*s. **a**, **c**, **e**, **g** and **i** represent the oil contents of the g1, g2, g3, g4, g123 and g134 lines, respectively. **b**, **d**, **f**, **h** and **j** represent the oil contents of the g1, g2, g3, g4, g123 and g134 lines, respectively. Data represent means ± SEs of three independent knock-out lines. Statistical differences between the wild type and mutants were determined by *t* test: *P < 0.05; **P < 0.01; the other differences are P ≥ 0.05). * in **b** represents the difference when excluding outliers

The oil contents of the mature seeds of the *Bnlpat2*/*Bnlpat5* mutant and wild type were measured to estimate whether *BnLPAT2* and *BnLPAT5* could influence oil accumulation. As expected, among all the transgenic lines obtained, the oil content in the gene knockout lines of *Bnlpat2* and *Bnlpat5* was significantly decreased (*P* < 0.01, ANOVA test), i.e., 35%, 30%, 31% and 29% decreases in oil content for g1, g2, g3 and g4, respectively (Fig. 7). Interestingly, a 39% decrease was detected in g134, but only a 24% decrease was detected in g123. These results revealed that the multi-gRNA vector might be more effective for multi-gene knockout, but not for single-gene knockout, which is the same as the efficiency to that of the multi-gene knockout above.

A significant change (*P* < 0.05, ANOVA test) in FA composition was also detected, especially for C18 and C20:0 FAs (Fig. 7). Specifically, in single-gRNA-mediated *Bnlpat2* mutant lines (i.e., g1, g2, g3), significant increases were detected in C18:0 and C20:0 with averages of 60% and 91%, whereas obvious decreases in C18:1, C18:2 and C18:3 were detected with averages of 8%, 8% and 21%, respectively. In g123 lines, a significant change was also detected with a 56% increase in C20:0; in addition, 11% decrease was observed in C18:2 and C18:33, respectively. Compared with the single-gRNA- and multi-gRNA-mediated *Bnlpat2* knockouts, the increases and decreases of FAs in g1/g2/g3 were greater than in g123. This result indicated that the CRISPR-Cas9 system is more effective in single-gRNA-mediated knockout than in multi-gRNA-mediated knockout when targeting only one gene. Moreover, in the *Bnlpat5* lines (i.e., g4), significant increases of 32% and 63% were detected in C18:0 and C20:0, while remarkable decreases of 14%, 6% and 11% in C18:1, C18:2 and C18:3 existed, respectively. In addition, in *Bnlpat2*/*Bnlpat5* double-mutated lines (i.e., g134), an almost 103% increase was observed in C20:0, whereas 5% and 23% decreases were observed in C18:2 and C18:3, respectively. This finding might demonstrate that the CRISPR-Cas9 system is more effective in multi-gRNA-mediated knockout than in single-gRNA-mediated knockout when targeting more than single gene.

## Discussion

Since its successful application in diploid plants, CRISPR-Cas9-system-mediated genome editing has brought about a revolution in polyploid plants. Precise editing at the genome level allows for crop modification with desired agronomic characteristics, such as disease resistance, herbicide tolerance and plant architecture modification [16, 34, 35]. Although the CRISPR-Cas9 system could mediate robust cleavage in many plants, there are still difficulties in polyploid plants for simultaneous multi-copy or multi-homologue knockout [25]. The present successful application in rapeseed will broaden the utilization of the CRISPR-Cas9 system for multigenic knockout in polyploid plants.

To date, several polyploid species have successfully achieved target gene modification by multi-homologue with CRISPR-Cas9 for desired morphological characters, including rapeseed, wheat, cotton, camelina and switchgrass [15, 26, 27, 36, 37]. For example, one sgRNA was constructed into a CRISPR-Cas9 backbone to successfully target two ALCATRAZ (ALC) homologues in tetraploid oilseed rape [25]. Two sgRNAs were designed to target the two copies of *GhCLA1* by CRISPR-Cas9 in the At and Dt subgenomes in allotetraploid cotton [36]. Two sgRNAs, which were initially designed from the closely related *Arabidopsis* FAD2 according to the website scoring criteria, were cloned into the CRISPR-Cas9 backbone to target three homoeologous FAD2 genes in hexaploid *Camelina sativa* [37]. It seems easier to knock out multiple homologues simultaneously in polyploid plants with no more than three homologues in nature. Thus, the sgRNAs were also easier to select by the scoring criteria offered by the CRISPR website. However, more than four copies generally exist in rapeseed for one gene, considering its genome polyploidy. Thus, more sgRNAs must be designed to knock out multiple copies simultaneously. In the present study, sgRNAs were deliberately designed from a highly conserved region, and seven paralogues of *BnLPAT*2 and four paralogues of *BnLPAT*5 were mutated simultaneously with CRISPR-Cas9, despite the comparatively low identities among these paralogues. In fact, only four paralogues of *BnLPAT*2 were searched from the *B. napus* genome database (http://www.genoscope.cns.fr/brassicanapus/). Another three copies (*Bna*A9*LPAT2*, *Bna*C8*LPAT2* and *Bna*A07*LPAT2*) were found with > 80% sequence identity with *Bna*A7*LPAT2* after 1 year, which might be because of the continuous updating, and they were considered part of the *BnLPAT2* family for later study. Fortuitously, the three sgRNAs (g1, g2 and g3) in *BnLPAT2* were exactly located in the latter three copies, respectively. Nevertheless, the three sgRNAs for *BnLPAT2* and the one sgRNA for *BnLPAT5* achieved multigenic mutations. In this way, it can avoid the utilization of multiple sgRNAs that must be designed individually and constructed together into one CRISPR-Cas9 backbone to target multiple homologous genes simultaneously, not only because of the difficulties in construction for up to five sgRNAs together, but also because of the further recombination of multiple sgRNAs themselves. Therefore, in the present study, the fewest sgRNAs were designed to knock out the largest number of desired genes. As expected, all the target genes were mutated with insertions, deletions or conversions in the target regions. To our knowledge, this is the most efficient and complete cleavage by CRISPR-Cas9 of one large complete gene family. The present results are encouraging for knocking out all the potential paralogues to obtain an obvious mutant, especially in polyploid plants.

Previously, 20 nt oligos (23 nt in total including NGG) have been designed for CRISPR-Cas9 knockout in rapeseed, rice or wheat [25, 32]. In the present study, 18 nt target sequences were deliberately designed, and effective gene editing in a context of simultaneous multigenic knockout was achieved. This revealed that the length of the target sequence could be loosened to 18–20 nt in rapeseed, which was also reported in rice [38]. Consequently, the shorter the target sequence, the greater is the convenience in target design for simultaneous multigenic knockout of all gene copies in polyploid plants. It also showed that the sgRNAs with moderate GC content (g2 and g4) achieved higher mutation efficiency than the lower—GC gRNAs (g1 and g3)—which was consistent with lower or higher G/C content (< 25%, > 75%) in sgRNAs tending to be lead to less activity while moderate-GC gRNAs (~ 50%) tend to be more active in CRISPR-Cas9-mediated knockout [39, 40]. Moreover, the sgRNA scaffold was commonly considered to be transcribed from A nucleotides by U3 promoters and G nucleotides by U6 promoters [41]. However, Ma et al. (2015) found that the regular targets (i.e., A for U3 and G for U6 promoter) and irregular targets (i.e., T or C for U3 and U6 promoter) showed similar editing efficiencies in monocotyledonous rice [42]. In the present study, among the three targets in *Bn*LPAT2, the first nucleotide of target 2 was initiated with G under the control of the *At*U6 promoter. Thus, g2 showed higher gene editing in *BnLPAT2*, which indicated that sgRNAs transcribed by the U6 promoter were effective when initiated with G nucleotides in dicotyledonous rapeseed. In addition, it showed a slight preference for the sense strand (g2) over the antisense strand (g1 and g3) in terms of sgRNA position, which was in contrast with the slight preference for the antisense strand previously observed by Wang et al. [40]. These differences might result from the lack of a statistically significant difference between the target strand and non-target strand [39].

Furthermore, unlike the limited mutant data in T0 plantlets reported by other researchers, a total of 100 mutant lines and 736 samples were evaluated to estimate gene editing efficiency with CRISPR-Cas9 in the present study. Similarly, it was found that the position of single base insertions in each target was located exactly 3 bp upstream of the PAM, which was consistent with previous reports [43, 44]. The present results revealed the high precision of CRISPR-Cas9-mediated cleavage system in rapeseed. Additionally, deletions varying from 1 to 53 bp, ignoring the PAM, were also observed as in other species, such as switchgrass, where − 1 bp to − 128 bp deletions occurred in target *tb1a* or *tb1b* [15]; Vu et al. designed two CRISPR-Cas9-induced cleavages between two tandem repeats at different sites, which resulted in 4.85% deletions of more than 1000 bp in target 1 and 3.2% deletions of > 1000 bp in target 2 in *A. thaliana*. The difference in the deletion fragment might be due to DNA sequence features, because the deletion size is closely related to the DSB repair profile [45]. These deletions of varied lengths ensured effective loss-of-function by CRISPR-Cas9-mediated knockout. Furthermore, all the substitutions observed in the present study were consistent with the conversions of cytidine deaminase-mediated mutations [33, 46]. These conversions are beneficial for achieving targeted conversion by CRISPR-Cas9-mediated mutations in the future. Notably, more than two types of mutations existed in one *Bnlpat2* or *Bnlpat5* plant. Generally, the genotype diversity would result in a chimaeric phenotype. This phenomenon might be due to cell diversity in the regenerated plantlets.

Simultaneous knockout of multiple genes is of great importance in polyploid plants. Because a single gRNA for one gene at a time makes it tedious to obtain a mutated plant with an obvious phenotype, the construction of multiple gRNAs in a CRISPR-Cas9 scaffold is frequently used to obtain more completely mutated plants. Compared to the single gRNA, multiple gRNAs could allow for knocking out multiple unrelated targets in different genes [30, 36]. However, it seems that the number and position of the sgRNAs would have an effect on gene editing efficiency. Previously, eight sgRNAs were constructed into the CRISPR-Cas9 binary vectors, but two target sites (*Os*FTL11 and *Os*02g0700600) showed no editing in rice T0 plants [42]. Owing to the positions of *Os*FTL11 and *Os*02g0700600, 7/8 and 3/3 corresponded to gene sequences, respectively. In addition, S2, S5 and S9 showed no editing with Cas9-mediated cleavage in rapeseed, as a result of the position, which was 2/2, 3/4 and 3/4 of the corresponding gene sequence [30]. In the present study, target 3 is in the position of 3/3 in *BnLPAT2*, and the cleavage ability is inactive in the g123 lines, but active in the g3 and g134 lines (Fig. 3a, Additional files 7, 8). The editing of target 3 in the g3 and g134 lines avoids this target sequence selectivity to guarantee uniform and reliable results in mutant lines. This revealed that the limited cleavage ability in g123 might owe to its hysteretic location in the gene sequence. Therefore, for large-scale CRISPR/Cas9 mutant library construction, the first two sgRNAs and an average of 2.59 sgRNAs were designed for each gene in rice, respectively [47, 48]. These also indicated that 2–3 sgRNAs might be a good choice for high-throughput mutant library research. Overall, the number and position of sgRNAs in multiple targets should be deliberately designed for high-efficiency gene editing. The results of the present study offer new powerful evidence for genome-wide mutant library construction using the CRISPR-Cas9 system.

High potential off-target sites with mismatches to the target were also checked. Among 14 potential off-target sites, none of these genes showed CRISPR-Cas9 system-induced cleavage as previously reported [16, 31, 49, 50]. In the present study, sgRNAs were deliberately designed for the entire family of *BnLPAT2* and *BnLPAT5* homologues. These results demonstrated that these four sgRNAs had a high specificity for CRISPR-Cas9-mediated gene editing in rapeseed. Some of this are due to the intentional plan to knockout the entire family of homologues, thus eliminating the most likely off-target sites. For example, in bread wheat, a single nucleotide mismatch is located at the cognate target site in *TaGW2*-*A1* compared to *TaGW2*-*B1* and -*D1*; thus, the mutagenesis frequencies for *TaGW2*-*B1* and -*D1* were 41.2% and 35.6%, respectively, whereas the off-target editing for *TaGW2*-*A1* was 30.8% by CRISPR-Cas9 in wheat protoplasts. However, no off-target mutations were detected in an additional set of 20 genomic sites with two to five nucleotide mismatches [51]. Furthermore, one sgRNA was designed to target two ALC homologues (*BnaA.ALC.a* and *BnaC.ALC.a*) with high sequence similarity (81%) and had one mismatch in *BnaC.ALC.a*. A mutation was detected in *BnaC.ALC.a*, while no mutation was detected in the other three putative off-target sites in the single obtained T0 plant in *B. napus* [25]. In addition, the sgRNA simultaneously targeted both copies of *BnCLV3*, which shared high sequence identity (90%), even though one mismatch was present in *BnC02.CLV3*. However, no mutations occurred in the 17 putative off-target sites in rapeseed [30]. These results might suggest that the off-target effect tends to occur in high-identity genes, which might most likely be highly homologous gene. That is, the off-target effect might be avoided by targeting the entire gene family of homologues. In addition, the latter three copies of *BnLPAT2* shared comparatively low sequence identity and presented no editing at the sites paired to the three targets of BnLPAT2 with one-to-two mismatches (Additional file 3 and Table 2). This result indicated that well-designed sgRNAs presented no editing at undesired sites in the present study. In other words, genome editing with the present CRISPR-Cas9-mediated knock out method is reliable in *B. napus*.

Functional redundancy is frequently observed in allotetraploid rapeseed, considering the two distinguishable but closely related homologous subgenomes in *B. napus*. The present study shows that CRISPR-Cas9 could efficiently knock out multiple gene copies simultaneously to obtain complete knockout mutants. Previously, it was found that a single-nucleotide mutation in a gene homologue of *BnCLV3* resulted in mutant siliques with 3–4 locules, while the double mutants of the *BnCLV3* homologous genes presented a full loss-of-function phenotype with 5.0–7.9 locules [30, 52]. To date, although LPAT has been characterized in several species, less is known in rapeseed [9, 53, 54]. No *Bnlpat* mutant has been reported using CRISPR-Cas9 technology owing to the multiple gene copies and homologues. However, overexpressing two rapeseed LPAAT isoforms (BAT1.13 and BAT1.5) in *Arabidopsis* seeds, a 16% significant increase was detected in mean total fatty acid content compared with the mean of the combined nontransformed plants [53]. Moreover, in the unicellular green microalga *Chlamydomonas reinhardtii*, overexpressing *CrLPAAT1* in plastids resulted to a > 20% increase in oil content under nitrogen-deficient conditions [10]. It seems that any increase in oil content is hard-won in model or simple organisms, let alone in allotetraploid rapeseed. In the present study, the multigenic knockout of *Bnlpat2* and *Bnlpat5* exhibited a significantly decreased oil content, with a 32% decrease in *Bnlpat2* lines, 29% in *Bnlpat5* lines and 39% in *Bnlpat2*/*Bnlpat5* double mutant lines. Therefore, simultaneous multigenic knockout by the CRISPR-Cas9 system has great potential in revealing gene function and generating agronomically important mutations in crops. Thus, the *Bnlpat2/Bnlpat5* mutants were generated by the CRISPR-Cas9 method, which provided the possibility to illuminate the mechanism of TAG synthesis. In the present study, the oil trait was affected by all the copies of each *BnLPAT* gene, and knockout of all the gene copies showed the desired phenotype, with an enlarged size of oil bodies and decreased oil content (Figs. 6, 7). Previously, the enlarged oil body phenotype was also found in *OLE1* and *OLE2* RNAi mutants [55]. Likewise, *pux10* knockout mutants also exhibited lipid droplets with increased size in *Arabidopsis* seedlings [56]. It considered that the enlarged oil bodies might owe to coalescence between neighboring oil bodies. In other words, *BnLPAT2*/*BnLPAT5* might play a role in preventing oil body fusion or perhaps contribute to oil body synthesis. In addition, the number of oil bodies also decreased in the mutant lines (Fig. 6), which was consistent with the phenomenon that the lipid droplet number was decreased, whereas an increase in size was observed in *Arabidopsis* lipid droplet-associated protein (*LDIP*) or *atsrp1* knock-down mutant [57, 58]. This result might indicate that *BnLPAT2*/*BnLPAT5* plays an important role in regulating the morphology and the number of oil bodies. Several studies have revealed a significant negative correlation between oil body size and oil content in *Arabidopsis* [59] and rapeseed [60]. The decrease in oil content in *Bnlpat2* and *Bnlpat5* mutants was negatively correlated with oil body morphology in the present study. According to the results of the average area for each oil body, it also showed that the larger the average size of the oil body is, the lower the oil content is (Additional file 9). In addition, the mutant rapeseed lines with a higher ratio of total oil bodies to cell area have been shown to have higher oil contents (Additional file 9). These inconsistent decreases might owe to the different substrate preferences of the two *BnLPAT*s in rapeseed [9, 53, 61]. The change in oil content offers powerful evidence that *BnLPAT2*/*BnLPAT5* play an important role in oil biosynthesis. They could possibly prevent the coalescence of normal oil bodies to form large oil bodies that increase the ratio of total oil bodies to cell area, which in turn might ultimately increase the total oil content. Additionally, a significant increase was detected in C18:0 and C20:0, while obvious decreases were detected in C18:1, C18:2 and C18:3 in the *Bnlpat2/Bnlpat5* lines in the present study, which was similar to the previous result that an increase in 18:1OH but a decrease in 20:1OH was observed when *Rc*LPAT2 was over expressed in *Lesquerella* [10]. This shows that the decrease in the final oil content was mostly derived from C18 and C20:0 FAs, which probably resulted in an increased conversion of FAs to other compounds, such as sucrose, in mature seeds. Collectively, the multigenic knockout of *BnLPAT2* and *BnLPAT5* might benefit future analyses of the regulation mechanism of oil content in rapeseed.

## Conclusion

In the present study, we demonstrated robust multi-locus genome editing with CRISPR-Cas9-mediated cleavage in allotetraploid rapeseed. Four single-gRNAs and two multiple-gRNAs were cloned into the CRISPR-Cas9-mediated mutation system; thus, seven *BnLPAT2* homologous genes and four *BnLPAT5* homologous genes were all completely cleaved, even though the sequence identity among these two gene families was relatively low. The transgenic efficiency varied from 82 to 100%, and the average transgenic efficiency was 93%, indicating that the transgenic system is very effective in rapeseed. Moreover, the mutation frequency for CRISPR-Cas9-mediated target gene cleavage in T0 the generation ranged from 17 to 68%. Insertions, deletions, substitutions and combined mutations were observed. Importantly, all the insertions occurred at the position of the third base pair proximal to the PAM region, whereas deletions (which varied from − 1 to − 53 bp) were not limited by the PAM, and some even crossed the PAM. Interestingly, we observed that target 3 was active in the g3 and g134 lines, but inactive in the g123 lines. This might benefit sgRNA selection for large-scale CRISPR-Cas9 mutations. In addition, no off-target cleavage was observed at the putative off-target sites. These results offered powerful evidence for effective gene editing with CRISPR-Cas9-mediated knockout in *B. napus*. Furthermore, increased oil body size and a significant decrease in oil content present in the T1 offspring of *Bnlpat2* and *Bnlpat5* mutant lines, which illustrated that multigenic knockout allowed a clear oil phenotype compared to single-gene overexpression or knockout. These results might contribute to the analysis of the regulatory mechanism of protein body formation and oil content biosynthesis in rapeseed and other species. Therefore, the high efficiency of the CRISPR-Cas9 technique will encourage more genome modification studies, especially in polyploid plants, such as mutant library construction or genes related to development and regulation. In summary, the prospects of the CRISPR-Cas9 technique in plants are encouraging, especially for polyploid crops with complex genomes.

## Methods

### Plant materials and growth conditions

The rapeseed genotype used in the present study is the semi-winter variety Jia2016, which was provided by Prof. Chunyu Zhang from Huazhong Agriculture University (Wuhan, China). All tissue culture was cultured at 25 °C with 3300 lx and 16 h light/8 h dark. The regenerated seedlings were vernalized at 4 °C for 2–4 weeks after development with 4–6 leaves. All the transgenic lines and WT lines were grown in the greenhouse at 24 °C with 2500 lx and 16 h light/8 h dark. During their growth, water was supplied three times a week. Aphids were controlled with imidacloprid (Jiangsu Changqing Biotechnology Co., China) and sticky coloured cards (Chunhe, China).

### Synteny, phylogenetic analysis and sequence alignment

The synteny analysis was performed according to the Syntenic Gene section of the online *Brassica* Database (http://brassicadb.org/brad/searchSyntenytPCK.php) and mapped by bioinformatics. Next, the complete protein sequences of LPAT2 and LPAT5 were retrieved from *Arabidopsis*, *B. oleracea*, *B. rapa* and *B. napus*. A phylogenetic tree was constructed using MEGA 7 software. Then, the gene sequences of *BnLPAT2* and *BnLPAT5* were extracted from the online *Brassica napus* Genome Database (http://www.genoscope.cns.fr/brassicanapus/). The sequence alignments were performed using VECTOR NTI software and exported as figures by Gene Doc.

### Vector construction for *Agrobacterium*-mediated transformation in *B. napus*

Sequence-specific targets for the pCRISPR-Cas9 plasmid were designed and constructed into the CRISPR-Cas9 vector by Shanghai GeneBio Co.,Ltd (Shanghai, China). The activity test in vitro was also performed by Shanghai GeneBio Co.,Ltd. These vectors were introduced into *Agrobacterium tumefaciens* GV3101 (WEIDI, Shanghai, China) via the electroporation method referring to the protocol with a small modification. Briefly, 10 μl of plasmid was added into 100 μl of *Agrobacterium* competent cells, and the mixture was electrically stimulated with 2.5 kV for 9 ms in the multi-function electric converter (Eppendorf, Germany). After that, the bacteria were grown in LB and stored at − 80 °C. The *Agrobacterium*-mediated transformation method for rapeseed was performed referred to previously with a little modification [62]. Briefly, the OD600 of the *Agrobacterium* strain reach to 1.6–2.0 in LB medium and 500 μl was gathered with a pipette for further infection. Plantlets with 4–6 leaves and well-developed roots were taken for further use.

### Identification of CRISPR-Cas9 mutants

Genomic DNA was extracted from the leaves of transgenic and WT plantlets according to the operation manual of the NuClean PlantGen DNA Kit (ComWin, Beijing, China). Then, primers were designed with OLIGO 7, and the transgenic-positive DNA samples were amplified to check the presence of a gRNA fragment with gRNA-F/R (Additional file 6). Next, to check the on-target mutation, approximately 700 bp DNA sequences that contained the target site from seven homologous *BnLPAT2* genes and the conserved region of four homologous *Bn*LPAT5 genes were amplified from independent lines using the specific primers listed in Additional file 6.

To further explore the in-dels of the target gene sequence, the PCR products were purified with QIAquick gel extraction kit (QIAGEN, USA) and were then cloned into the pMD18-T vector (TaKaRa, Japan). After transformed into *E. coli*, ten positive single colonies for each plate were randomly picked and identified by PCR amplification for Sanger sequencing.

### Microscopy analysis and seed morphology scanning

For TEM, cotyledons were isolated from dry seeds and were then fixed in 2.5% glutaraldehyde. The follow-up work was performed at Servicebio Co., Ltd (Wuhan, China). The area of each oil body was traced with IMAGE J software for each line. The results of the oil body area were multiplied by 100, transformed with Log2, and then evaluated with the online software E Chart (http://www.ehbio.com/ImageGP/). Three duplicates were examined for each mutant line.

For seed scanning, mature dry seeds were weighed with an analytical balance (Mettler Toledo, Changzhou, China), and then all seeds were scanned using SC-G automatic seed testing and thousand-particle weight analyser (Wseen, Co., Ltd, Hangzhou, China).

### Oil extraction and fatty acid analysis

Total oil was extracted from dried mature seeds according to the methods published previously [63]. In detail, approximately 300 mg seeds were weighed, and 2.5% H_2_SO_4_-methyl alcohol, C17:0-methylbenzene and methylbenzene were added in order, After 1 h for 90 °C, double distilled water and hexyl hydride were added, the mixture was incubated for 12 h, and the liquid supernatant was collected. FA analysis was performed using gas chromatography with Agilent 7890A as according to the standard procedure. Triplicates were independently performed for each line in our study, and mean values of the triplicates were used for calculation as described previously [64]. Relative FA compositions were calculated as the percent of each FA relative to the total measured.

## Supplementary information

**Additional file 1.** Sequence alignment of the four BnLPAT2 homologous genes.

**Additional file 2.** Sequence alignment of the four BnLPAT5 homologous genes.

**Additional file 3.** Homology matrix of seven homologous BnLPAT2 genes and four homologous BnLPAT5 genes.

**Additional file 4.** In vitro assay of three guide RNAs mediated Cas9 activity. The arrow represents the digested fragments. M, 1000 bp DNA marker. S-1 and S-2 mean standard gRNA, whose SSA activity is 30% and 100%, respectively.

**Additional file 5.** PCR detection gene insertion in the T0 generation. Cropped gel image showing the PCR products of gRNA-sac in different progeny from g1, g2, g3 and g4 lines. Marker, DL 2000 bp. −: gDNA from WT was used as a negative control.

**Additional file 6.** Primers and their purpose used in this study.

**Additional file 7.** Single-gRNA mediated target for knockout by CRISPR-Cas9 mediated cleavage. “−” and “+” indicate nucleotide insertions and deletions in the target sequence. Red indicates indels in the target sequence. gRNAs are coloured yellow, whereas PAMs are coloured green.

**Additional file 8.** Multi-gRNA mediated target for knockout by CRISPR-Cas9 mediated cleavage. “−” and “+” indicate nucleotide insertions and deletions in the target sequence. Red indicates the indels in the target sequence. gRNAs are coloured in yellow, whereas PAMs are coloured in green.

**Additional file 9.** The area ratio of total oil bodies and the average size of each oil body.

## Data Availability

Additional files 1, 2, 3, 4, 5, 6, 7, 8, 9: additional material to “Effective editing of lysophosphatidic acid acyltransferase 2/5 in allotetraploid rapeseed (*Brassica napus* L.) using CRISPR-Cas9 system”.

## Abbreviations

LPAT: lysophosphatidic acid acyltransferase
CRISPR-Cas9: clustered regularly interspaced short palindromic repeats—CRISPR-associated proteins 9
PAM: protospacer adjacent motif
g1, g2 and g3: three different single-gRNA-mediated *Bnlpat2* knockout lines, respectively
g4: single-gRNA-mediated *Bnlpat5* knockout line
g123: multi-gRNA (i.e., g1, g2 and g3)-mediated *Bnlpat2* knockout line
g134: multi-gRNA (i.e., g1, g3 and g4)-mediated *Bnlpat2*/*Bnlpat5* knockout line
TAG: triacylglycerols
OFF: off target
FA: fatty acid
LPA: lysophosphatidic acid
PC: phosphatidylcholine
PE: phosphatidylethanolamine
