# Supplementary material for: Effective editing for lysophosphatidic acid acyltransferase 2/5 in allotetraploid rapeseed (Brassica napus L.) using CRISPR-Cas9 system
Source: Biotechnol Biofuels. 2019 Sep 20;12:225. doi: 10.1186/s13068-019-1567-8 (PMC6753616; doi:10.1186/s13068-019-1567-8)
Supplement: Supplementary file 1 — Additional file 1. Sequence alignment of the four BnLPAT2 homologous genes. [file 13068_2019_1567_MOESM1_ESM.pdf]

```
                   *        20         *        40         *        60         *        80         *       100         *       120         *
BnLPAT2-C4 : ATGGCGATGGCTGCAGCTG---TAATCGTGCCTTTAGGCCTTCTCTTCTTCATCTTTGGTCTCCTCGTGAACCTCCTCCTAGCAATTTGCTA--T----------------------------------- :  90
BnLPAT2-A4 : ATGGCGATAACTGCAGCTG---TAATAGTGCCTTTAGGCCTTCTCTTCTTCATCTCTGGTCTCCTCATGAACCTCCTTCAGGCAATTTGTTATGTACTCGTTAGACCTCTGTCTAAGAACACATACAGAA : 127
BnLPAT2-A7 : ATGGCGATGGCAGCAGCAG---TGATTGTGCCTTTGGGGATTCTCTTCTTCATTTCTGGCCTCGTTGTCAATCTCCTTCAGGCAGTTTGCTATGTCCTCGTTCGACCTCTGTCTAAGAACACATACAGAA : 127
BnLPAT2-C7 : ATGGCGATGGCAGCAGCAGCAGTGATTGTGCCTTTGGGGATTCTCTTCTTCATTTCTGGCCTCGTTGTCAATCTCCTTCAGGCAGTTTGCTATGTCCTCATTCGACCTCTGTCTAAGAACACATACAGAA : 130
BnLPAT2-A9 : ATGGCGATGGCAGCAGCTG---TGATTGTGCCTCTGGGAATTCTCTTCTTCATATCTGGTCTCGTTGTCAATCTCCTTCAGGCGATTTGTTATGTTCTTATTCGACCTCTGTCTAAGAACACGTACAGAA : 127
BnLPAT2-C8 : ATGGCGATGGCAGCAGCTG---TGATTGTGCCTCTGGGCATTCTCTTCTTCATATCTGGTCTCGTTGTTAATCTCCTTCAGGCGATTTGTTATGTTCCTATTCGACCTCTGTCTAAGAACACGTACAGAA : 127
BnLPAT2-A07: ---------------------------------------------------------------------------------------------------------------------------------- :  -
             atggcgat  c gcagc g   t at gtgcct t gg  ttctcttcttcat t tgg ctc t  t aa ctcct c  gc  tttg ta  t

                140         *       160         *       180         *       200         *       220         *       240         *       260
BnLPAT2-C4 : ------------------C--------------------TTGTCTGGATCGTTGACTGGTGGGCAGGAGTCACGATCAAAGTCTTTACTGATAATGAGACGTTTAATCGAATGGGCAAAGAACACGCTCT : 182
BnLPAT2-A4 : AAATCAACCGGGTGGTCGCAGAAACTTTGTGGCTTGAGCTTGTCTGGATCGTTGACTGGTGGGCAGGAGTCAAGATCAAAGTGTTTGCTGATAATGAGACCTTCAGTCGAATGGGAAAAGAACATGCTCT : 257
BnLPAT2-A7 : AGATCAACCGGGTGGTTGCAGAAACCTTGTGGTTGGAGCTTGTCTGGATCGTTGACTGGTGGGCTGGAGTCAAGATCCAAGTCTTTGCTGATGATGAGACCTTTAATCGAATGGGCAAAGAACATGCTCT : 257
BnLPAT2-C7 : AGATCAACCGTGTGGTTGCAGAAACCTTGTGGTTGGAGCTTGTCTGGATCGTTGACTGGTGGGCTGGAGTCAAGATCCAAGTGTTTGCTGATGATGAGACCTTTAATCGAATGGGCAAAGAGCATGCTCT : 260
BnLPAT2-A9 : AAATCAACCGGGTGGTTGCTGAAACCTTGTGGCTTGAGCTTGTCTGGATTGTTGACTGGTGGGCTGGTGTAAAGATCCAAGTGTTTGCTGATAATGAGACCTTCAATCGAATGGGCAAAGAACATGCTCT : 257
BnLPAT2-C8 : AAATCAACCGGGTGGTTGCTGAAACCTTGTGGCTTGAGCTTGTCTGGATTGTTGACTGGTGGGCTGGTGTAAAGATCCAAGTGTTTGCTGATAATGAGACCTTCAATCGAATGGGCAAAGAACATGCTCT : 257
BnLPAT2-A07: ---------------------------------------------------------------------------------------------------------------------------------- :  -
                           c                   ttgtctggat gttgactggtgggc gg gt a gatc aagt ttt ctgat atgagac tt a tcgaatggg aaaga ca gctct

                   *       280         *       300         *       320         *       340         *       360         *       380         *
BnLPAT2-C4 : TGTCGTTTGTAATCACCGAAGTGATATTGATTGGCTTGTGGGATGGATTCTCGCTCAGAGGTCAGGTTGCCTGGGAAGCGCATTAGCTGTAACGAAGAAGTCTTCCAAATTTCTTCCAGTCAAAGGCTGG : 312
BnLPAT2-A4 : TGTCGTTTGTAATCACCGAAGTGATATCGATTGGCTTGTGGGATGGATTCTGGCTCAGAGGTCAGGTTGCCTGGGAAGTGCGTTAGCTGTAACGAAGAATTCTAACAAGTTTCTTCCAGTCATAGGCTGG : 387
BnLPAT2-A7 : TGTCGTTTGTAATCACCGAAGTGATATTGATTGGCTCGTGGGATGGATTCTCGCTCAGAGGTCAGGTTGCCTAGGAAGCGCATTAGCTGTGATGAAGAAGTCTTCCAAATTTCTCCCAGTCATAGGCTGG : 387
BnLPAT2-C7 : TGTCGTTTGTAATCACCGAAGTGATATTGATTGGCTCGTGGGATGGATTCTGGCTCAGAGGTCAGGTTGCCTAGGAAGCGCATTAGCTGTGATGAAGAAGTCTTCCAAATTTCTTCCAGTCATAGGCTGG : 390
BnLPAT2-A9 : TGTCGTTTGTAATCACCGAAGTGATATTGATTGGCTTGTGGGATGGATTCTGGCTCAGAGATCAGGTTGCCTGGGAAGCGCATTGGCTGTAATGAAGAAGTCTTCTAAATTTCTTCCAGTCATAGGCTGG : 387
BnLPAT2-C8 : TGTCGTTTGTAATCACCGAAGTGATATTGATTGGCTTGTGGGATGGATTCTGGCTCAGAGATCAGGTTGCCTGGGAAGCGCATTGGCTGTAATGAAGAAGTCTTCTAAATTTCTTCCAGTCATAGGCTGG : 387
BnLPAT2-A07: ---------------------------------------------------------------------------------------------------------------------------------- :  -
             tgtcgtttgtaatcaccgaagtgatat gattggct gtgggatggattct gctcagag tcaggttgcct ggaag gc tt gctgt a gaagaa tct   aa tttct ccagtca aggctgg

                400         *       420         *       440         *       460         *       480         *       500         *       520
BnLPAT2-C4 : TCAATGTGGTTCTCAGAGTATCTATTTCTGGAAAGAAACTGGGTAAAGGATGAAAGCACAATAAAGTCAGGTCTTCAACGCTTGAAAGACTTCCCTCAGCCCTTCTGGCTAGCCCTTTTTGTGGAGGGAA : 442
BnLPAT2-A4 : TCAATGTGGTTCTCAGAGTTTCTGTTTTGGAAAGAAATTGGGCAAAGGATGAAAGCACAATAAAGTCAGGTCTTCAACGCTTGAAAGACTTCCCTCAGCCTTTCTGGCTAGCCCTTTTTGTGGAGGGAA : 517
BnLPAT2-A7 : TCAATGTGGTTCTCCGAGTATCTGTTTCTTGAAAGAAATTGGGCAAAGGATGAAAGCACTTTAAAGTCAGGTCTTCAACGCTTGAACGACTTCCCACGGCCTTTCTGGCTAGCTCTTTTTGTGGAGGGAA : 517
BnLPAT2-C7 : TCAATGTGGTTCTCGGAGTATCTGTTTCTCGAAAGAAATTGGGCAAAGGATGAAAGCACTTTAAAGTCAGGTCTTCAACGCTTGAACGACTTCCCACGGCCTTTCTGGTTAGCCCTTTTTGTGGAGGGAA : 520
BnLPAT2-A9 : TCAATGTGGTTCTCGGAGTATCTGTTTCTGGAAAGAAATTGGGCAAAGGATGAAAGCACTCTAAAGTCAGGTCTTCAACGCTTGAACGACTTCCCTAGACCTTTCTGGTTAGCACTTTTTGTGGAGGGAA : 517
BnLPAT2-C8 : TCAATGTGGTTCTCGGAGTATCTGTTTCTGGAAAGAAATTGGGCAAAGGATGAAAGCACTCTAAAGTCAGGTCTTCAACGCTTGAACGACTTCCCTAGACCTTTCTGGTTAGCACTTTTTGTGGAGGGAA : 517
BnLPAT2-A07: --------------------------------------ATGTGTGTTTATCCAAATCA----ACAGTCAGGTCTTCAACGCTTGAACTACTTCCCACGTCCTTTCTGGTTAGCCCTTTTTGTTGAGGGGA :  88
             tcaatgtggttctc gagt tct ttt t gaaagaaa TGgG aaaggatgAAAgCAc  tAaAGTCAGGTCTTCAACGCTTGAA gACTTCCC    CCtTTCTGG TAGC CTTTTTGTgGAGGGaA

                   *       540         *       560         *       580         *       600         *       620         *       640         *
BnLPAT2-C4 : CTCGCTTTACAGAGACGAAACTTAAAGCAGCACAAGAGTACGCAGCTTCCTCTGAGTTGCCTATCCCTCGAAATGTCTTGATTCCTCGTACCAAAGGTTTTGTGTCAACAGTTATTAATATGCGTTCATT : 572
BnLPAT2-A4 : CCCGCTTTACAGAGACAAAACTTAAAGCAGCACAAGAATACGCAGCGTCCTCTGAGTTGCCTATCCCTCGTAATGTCTTGATTCCTCGTACCAAAGGTTTTGTGTCAACGGTAAGTAATATGCGTTCATT : 647
BnLPAT2-A7 : CCCGCTTCACAGAGGCAAAACTTAAAGCAGCACAAGAGTACGCAGCCTCCTCTGAGTTGCCTGTCCCTCGAAATGTGTTGATTCCTCGCACCAAAGGATTTGTGTCAGCTGTTAGTAACATGCGTTCATT : 647
BnLPAT2-C7 : CCCGTTTCACAGAGGCAAAACTTAAAGCAGCACAAGAGTACGCAGCCTCCTCTCAGTTGCCTGTCCCTCGAAATGTGTTGATTCCTCGCACCAAAGGATTTGTGTCAGCTGTTAGTAACATGCGTTCATT : 650
BnLPAT2-A9 : CCCGCTTTACAGAGGCTAAACTTAAAGCAGCACAAGAGTACGCTGCCTCCTCTGAGCTGCCTGTCCCTCGAAATGTGTTGATTCCTCGCACCAAAGGTTTTGTGTCAGCTGTTAGTAATATGCGTTCATT : 647
BnLPAT2-C8 : CCCGCTTTACAGAGGCTAAACTTAAAGCAGCACAAGAGTACGCTGCCTCCTCTGAGCTGCCTGTCCCTCGAAATGTGTTGATTCCTCGCACCAAAGGTTTTGTGTCAGCTGTTAGTAATATGCGTTCATT : 647
BnLPAT2-A07: CCCTCTTTACTGAGGCAAAACTTAAAG--T--------GTACGCAGCCTCCTCTGAGTTGCCTATCCCTCGAAATGTGTTGATTCCTCGCGCCAAAG--TAAGTTCATAAGTT--T-----TCTTTCTTT : 200
             CcCgcTT ACaGAG C AAACTTAAAGcagcacaagagTACGC GC TCCTCTgAG TGCCT TCCCTCGaAATGT TTGATTCCTCG aCCAAAGg TttGTgTCA c GTta Taa atgCgTTCaTT

                660         *       680         *       700         *       720         *       740         *       760         *       780
BnLPAT2-C4 : TGTCCAGTCATTTATGATATGACCGTGGCTATTCCAAAATCTTCTCCTCCCCCAACAATGCTAAGACTATTCAAAGGACAACCTTCATT-------------ATCCATTTTCTTTACATTAGAAATTTT : 689
BnLPAT2-A4 : TGTCCAGCCATTTATGATATGACCGTGGCTATTCCAAAAACTTCTCCACCCCCAACAATGCTAAGACTATTCAGAGGACAGCCTTCTGTGGTGCATGTTCACATAAAGTGTCACTCGATGAAAGACTTG : 777
BnLPAT2-A7 : TGTGCCAGCCATATATGATATGACCGTGGCTATTCCAAAAACTTCTCCACCCCCAACGATGCTAAGACTATTCAAAGGACAACCTTCTGTGGTGCATGTTCACATCAAGTGTCACTCGATGAAAGACTTG : 777
BnLPAT2-C7 : TGTGCCAGCCATATATGATATGACCGTGGCTATTCCAAAAACTTCTCCACCCCCAACGATGCTAAGACTATTCAAAGGACAACCTTCTGTGGTGCATGTTCACATCAAGTGTCACTCGATGAAAGACTTG : 780
```

```
BnLPAT2-A9 : TGTCCCAGCCATTTATGATATGACCGTGGCTATTCCAAAAACATCTCCACCCCCAACGATGCTCAGACTATTCAAAGGACAACCTTCTGTGGTGCATGTTCACATCAAGTGTCACTCGATGAAAGACTTG :  777
BnLPAT2-C8 : TGTCCCAGCCATTTATGATATGACCGTGGCTATTCCAAAAACATCTCCACCCCCAACGATGCTCAGACTATTCAAAGGACAACCTTCTGTGGTGCATGTTCACATCAAGTGTCACTCGACGAAAGACTTG :  777
BnLPAT2-A07: TG-CACATTCCTTGGGCAACTTGCTCTCCTCACTG------------------------------------------------------------------------------------------------------------------- :  234
             TGt cCAg CaT tatgAtaTgaCcgTggctAtTccaaaa c tctcc cccccaac atgct agactattca aggaca ccttc  t          at  a t tc  t  a  a a a tt

                     *      800         *      820         *      840         *      860         *      880         *      900         *
BnLPAT2-C4 : C---ATTCTG--GA--------CACACTCAT-CTCATCTCGGCTCTGTGGTACAGGATGCACTGTTAGACAAACACATAGCGGCAGATACTTTCTCCCATCAGCAAGAACAGAACATTGGCCGTCCCATG :  805
BnLPAT2-A4 : CCTGAATCAGATGAGGCAATTGCACAGTGGTGCATAAATCAGTT-TGTGGCTAAGGATGCACTGTTAGACAAACACATAGCGGCAGACACTTTCCCTGGTCAGCAGGAGCAGAACATTGGCCGTCCCATA :  906
BnLPAT2-A7 : CCTGAATCAGAAGACGAAATTGCACAGTGGTGCAGAGATCAGTT-TGTGACTAAGGATGCACTGTTAGACAAACACATAGCTGCAGACACTTTCGCCGGTCAGAAAGAACAGAACATTGGCCGTCCCATA :  906
BnLPAT2-C7 : CCTGAATCAGAAGACGAAATTTCACAGTGGTGCAGAGATCAGTT-TGTGGCTAAGGATGCACTGTTAGACAAACACATAGCTGCAGACACTTTCCCCGGTCAGAAAGAACAGAACATTGGCCGTCCCATA :  909
BnLPAT2-A9 : CCTGAATCAGATGACGCAATTGCACAGTGGTGCAGAGATCAGTT-TGTGGCTAAGGATGCACTATTAGACAAACACATAGCTGCAGACACTTTCCCTGGTCAGCAAGAACAGAACATTGGCCGTCCCATA :  906
BnLPAT2-C8 : CCTGAATCAGATGACGCAATTGCACAGTGGTGCAGAGATCAGTT-TGTGGCTAAGGATGCACTATTAGACAAACACATAGCTGCAGACACTTTCCCTGGTCAGCAAGAACAGAACATTGGCCGTCCCATA :  906
BnLPAT2-A07: ------------------------------------------------------------------------------------------------------------------------------------------------------ :   -
             c   a tc g  ga       caca  t  t c  a  tc g  t tgtg    aggatgcact ttagacaaacacatagc gcaga actttc c   tcag a ga cagaacattggccgtcccat

                 920         *      940         *      960         *      980         *     1000         *     1020         *     1040
BnLPAT2-C4 : TAGTCTCTTGCAGTGGTTCTATCATGGGCCTGCATACTAACTCTCGGAGCAATGAAATTCTTGCACTGGTCAAATCTCTTTTCCTCATGGAAAGGCATGGCGTTATCAGCGCTTGGTCTAGGAAGATTAC :  935
BnLPAT2-A4 : AAGTCTCTTGCAGTGGTTTTATCATGGGCCTGCCTACTAACTCTAGGAGCAATTAAATTCTTACACTGGTCAGATCTCTTTTCCTCATGGAAAGGCATGGCGTTGTCGGCACTTGGTCTAGGTGTCAT-C : 1035
BnLPAT2-A7 : AAGTCTCTTGCAGTGGTTCTGTCATGGGCATGTCTACTAACTCTTGGAGCAATGAAGTTCTTACACTGGTCAAATCTCTTTTCCTCGTGGAAAGGCATCGCATTATCAGCGCTTGGTCTAGGCATCAT-C : 1035
BnLPAT2-C7 : AAGTCTCTTGCAGTGGTTGTATCATGGGCATGCCTACTAACTCTTGGAGCAATGAAGTTCTTACACTGGTCAAATCTCTTTTCCTCGTTGAAAGGCATCGCATTATCAGCGCTTGGTCTAGGCATCAT-C : 1038
BnLPAT2-A9 : AAGTCTCTTGCAGTGGTTCTATCATGGTCATGCCTACTGATTCTTGGAGCAATGAAGTTCTTACACTGGTCAAATCTCTTCTCCTCATGAAAAGGCATCGCGTTTTCGGCGCTGGGTCTAGGCATCAT-C : 1035
BnLPAT2-C8 : AAGTCTCTTGCAGTGGTTCTATCATGGTCATGCCTACTGATTCTTGGAGCAATGAAGTTCTTACACTGGTCAAATCTCTTCTCCTCATGGAAAGGCATCGCGTTTTCGGCGCTGGGTCTAGGCATCAT-C : 1035
BnLPAT2-A07: ------------------------------------------------------------------------------------------------------------------------------------------------------ :   -
              agtctcttgcagtggtt t tcatgg c tg  tact a tct ggagcaat aa ttctt cactggtca atctctt tcctc t  aaaggcat gc tt tc gc ct ggtctagg    t c

                     *     1060         *     1080         *     1100         *     1120         *     1140         *     1160         *
BnLPAT2-C4 : GGTAGCAGATATTATAAAGTG-TGCGAAAGTGTCTGTAATACCATTCGCAGTTCATATGGGTGAAGTTTGTAGTGTTCACAAGCTTAAAATAAATCTCTATAAGCCAGAGGATTATCCTAAGCACACTGA : 1064
BnLPAT2-A4 : ACTCTCTGTATGCAGATCCTGATCCGTTCCTCTCAGTCAGAGCGTTGG------ACCC-----------------CAGCCAAGACAAATGACCATC---ACCACGCAG--GATCATTCT-----CCCAAA : 1132
BnLPAT2-A7 : ACTCTCTGTATGCAGATCTTGATCCGCTCCTCTCAGTCGGAGCGTTCA------ACACCTGCCAAAGTCGC--TCCAGCCAAGCCAAAGGACAATC---ACCAGTCAG--GACCATCCT-----CCCAAA : 1147
BnLPAT2-C7 : ACTCTCTGCATGCAGATCTCGATCCGCTCCTCTCAGTCGGAGCGTTCA------ACACCTGCCAAAGTGGC--CCCAGCCAAGCCAAAGGACAAAC---ACCAGTCAG--GATCATCCT-----CCCAAA : 1150
BnLPAT2-A9 : ACTCTCTGTATGCAGATCCTGATCCGTTCCTCTCAGTCAGAGCGTTCT------ACCCCAGCCAAAGTCGT--TCCAGCCAAGCCAAAAGACAATCATAACGACTCAG--GATCATCCT-----CCCAAA : 1150
BnLPAT2-C8 : ACTCTCTGTATGCAGATCCTGATCCGTTCCTCTCAGTCAGAGCGTTCT------ACCCCAGCCAAAGTCGT--TCCAGCCAAGCCAAAAGACAATCATAACGACTCAG--GATCATCCT-----CCCAAA : 1150
BnLPAT2-A07: ------------------------------------------------------------------------------------------------------------------------------------------------------ :   -
               t  c g     a a    g t cg    t tc gt   a c tt        a                     caag   aa     a c   a  a  cag   ga  at ct     c c  a

                1180         *     1200         *     1220         *     1240         *     1260         *     1280         *     1300
BnLPAT2-C4 : TATAACTGAAGCACACTTGGAAGAGACAATGACGGTTATGTTTTTGAGGAAGTTGGAAGATGCTGAGCATGCTATATTTATTTTTGCAGCTTATGCAAGTCATATTACTGATAAGTTTGAGGAAGATTAC : 1194
BnLPAT2-A4 : CAGATGTGGAG---AAGGACAAG------------------------------------------------------------------------------------------------------------------------------- : 1152
BnLPAT2-A7 : CAGAAGTGGAGGAGAAGCAGAAGTAA---------------------------------------------------------------------------------------------------------------------------- : 1173
BnLPAT2-C7 : CAGAAGTGGAGGAGAAGCAGAAGTAA---------------------------------------------------------------------------------------------------------------------------- : 1176
BnLPAT2-A9 : CAGAAGTGGAG---AAGCAGAAGTAA---------------------------------------------------------------------------------------------------------------------------- : 1173
BnLPAT2-C8 : CAGAAGTAGAG---AAGCAGAAGTAA---------------------------------------------------------------------------------------------------------------------------- : 1173
BnLPAT2-A07: ------------------------------------------------------------------------------------------------------------------------------------------------------ :   -
             a a  t  ag   a     aag

                     *     1320         *     1340         *     1360         *     1380         *     1400         *     1420         *
BnLPAT2-C4 : GGTAGCAGATATTATAAAGATTATGGAAGTGTCCGTAGCAGTTCAATGAGCCGAGGCCAAGGGTACGGAGGCGGGGTTGATCTTGATGCAGATGCACAGAAACGTAAAAAGCAAGCAACAGATGAGCAGG : 1324
BnLPAT2-A4 : ------------------------------------------------------------------------------------------------------------------------------------------------------ :   -
BnLPAT2-A7 : ------------------------------------------------------------------------------------------------------------------------------------------------------ :   -
BnLPAT2-C7 : ------------------------------------------------------------------------------------------------------------------------------------------------------ :   -
BnLPAT2-A9 : ------------------------------------------------------------------------------------------------------------------------------------------------------ :   -
BnLPAT2-C8 : ------------------------------------------------------------------------------------------------------------------------------------------------------ :   -
BnLPAT2-A07: ------------------------------------------------------------------------------------------------------------------------------------------------------ :   -


                1440         *     1460         *     1480         *     1500         *     1520         *     1540         *     1560
```

```
BnLPAT2-C4 : GTTATGAAGAAAGTTGTGACGATGAGAAGAACGAACCATCGTTCACTGGTGGAGTAGATGACCTTGTGATGGATAATGAGGATGAGAACACAGAAGTGGCTTATTGTGGTGATCCTCAAGTGATATGGGA : 1454
BnLPAT2-A4 : ---------------------------------------------------------------------------------------------------------------------------------- :    -
BnLPAT2-A7 : ---------------------------------------------------------------------------------------------------------------------------------- :    -
BnLPAT2-C7 : ---------------------------------------------------------------------------------------------------------------------------------- :    -
BnLPAT2-A9 : ---------------------------------------------------------------------------------------------------------------------------------- :    -
BnLPAT2-C8 : ---------------------------------------------------------------------------------------------------------------------------------- :    -
BnLPAT2-A07: ---------------------------------------------------------------------------------------------------------------------------------- :    -

                  *     1580        *
BnLPAT2-C4 : AGAGTTGCACAATCAACTTCTCAATTCCGCAGAA : 1488
BnLPAT2-A4 : ---------------------------------- :    -
BnLPAT2-A7 : ---------------------------------- :    -
BnLPAT2-C7 : ---------------------------------- :    -
BnLPAT2-A9 : ---------------------------------- :    -
BnLPAT2-C8 : ---------------------------------- :    -
BnLPAT2-A07: ---------------------------------- :    -
```
