# Supplementary material for: Effective editing for lysophosphatidic acid acyltransferase 2/5 in allotetraploid rapeseed (Brassica napus L.) using CRISPR-Cas9 system
Source: Biotechnol Biofuels. 2019 Sep 20;12:225. doi: 10.1186/s13068-019-1567-8 (PMC6753616; doi:10.1186/s13068-019-1567-8)
Supplement: Supplementary file 2 — Additional file 2. Sequence alignment of the four BnLPAT5 homologous genes. [file 13068_2019_1567_MOESM2_ESM.pdf]

```
                      *        20         *        40         *        60         *        80         *       100         *       120         *
BnLPAT5-A5 : ATGATGTTGATTTTCTGGGGGTTCCTATCAGCTGTAGTGATGAGGCTTTTCAGCTTACACTACAGCCGTAAATGTGTTTATTTCTTCTTTGGCTCCTGGCTCGCCTTGTGGCCTTTCCTCTTTGAGAAGA :  130
BnLPAT5-C5 : ATGATGTTGATTTTCTGGGGGTTCCTATCAGCTGTAGTGATGAGGCTTTTCAGCTTACACTACAGCCGTAAATGTGTTTATTTCTTCTTTGGCTCGTGGCTCGCCTTGTGGCCTTTCCTCTTTGAGAAGA :  130
BnLPAT5-C1 : ATGATGTTGATATTCTGGGGGTTCCTATCAGGTGTAGTGTTGAGACTCTTCAGCATACACTATAGCCGTAAATGTGTTTCTTTCTTCTTCGGCTCCTGGCTCGCCTTGTGGCCTTTCCTCTTTGAGAAGA :  130
BnLPAT5-UK : ATGATGTTGATATTCTGGGGGTTCCTATCAGGCGTAGTGTTGAGACTCTTCAGCATACACTATAGCCGTAAATGTGTTTCTTTCTTCTTCGGCTCCTGGCTCGCCTTGTGGCCTTTCCTCTTTGAGAAGA :  130
             ATGATGTTGAT TTCTGGGGGTTCCTATCAG tGTAGTG TGAG CT TTCAGC TACACTA AGCCGTAAATGTGTTT TTTCTTCTT GGCTCcTGGCTCGCCTTGTGGCCTTTCCTCTTTGAGAAGA

                  140         *       160         *       180         *       200         *       220         *       240         *       260
BnLPAT5-A5 : TCAATAGAACCAAAGTTATCTTCTCAGGGGATAAGGTTCCTTGTGAGTCACGAGTGTTGCTCATTGCCAATCACCGAACAGAGGTTGACTGGATGTACTTCTGGGATCTTGCACTGCGTAAAGGTCAGAT :  260
BnLPAT5-C5 : TCAACAGAACCAAAGTTATCTTCTCTGGGGATAAGGTTCCTTGTGAGTCACGAGTGTTGCTCATTGCCAATCACCGAACAGAGGTTGACTGGATGTACTTCTGGGATCTTGCACTGCGTAAAGGTCAGAT :  260
BnLPAT5-C1 : TAAACGGAACCAAAGTTGTCTTCTCTGGGGATAAAGTTCCTTGCGAGGAACGAGTATTGCTCATTGCGAATCACCGAACAGAAGTTGATTGGATGTACTTCTGGGATCTTGCACTGCGCAAAGGCCAGAT :  260
BnLPAT5-UK : TAAACGGAACCAAAGTTGTCTTCTCTGGGGATAAAGTTCCTTGCGAGGAACGAGTATTGCTCATTGCGAATCACCGAACAGAAGTTGATTGGATGTACTTCTGGGATCTTGCACTGCGCAAAGGCCAGAT :  260
             T AAc GAACCAAAGTT TCTTCTCtGGGGATAA GTTCCTTG GAG  ACGAGT TTGCTCATTGC AATCACCGAACAGA GTTGA TGGATGTACTTCTGGGATCTTGCACTGCG AAAGG CAGAT

                      *       280         *       300         *       320         *       340         *       360         *       380         *
BnLPAT5-A5 : TGGGAATATGAAGTATGTCCTTAAGAGCAGTCTGATGAAGTTGCCTCTGTTCGGTTGGGCGTTTCACCTCTTTGAGTTTATCCCCGTTGAGCGGAAATGGAAAGTAGATGAAGCAAACTTGAGGCAGATG :  390
BnLPAT5-C5 : TGGGAATATGAAGTATGTCCTTAAGAGCAGTCTGATGAAGTTGCCTCTGTTCGGTTGGGCGTTTCACCTCTTTGAGTTTATCCCCGTTGAGCGGAAATGGAAAGTAGATGAAGCAAACTTGAGGCAGATG :  390
BnLPAT5-C1 : TGGGAACATGAAGTATGTGCTTAAGAGCAGCTTGATGAAACTACCTCTGTTTGGTTGGGCGTTTCACCTCTTTGAGTTTATTCCCGTTGAGCGGAGATGGGAAGTGGATGAAGCGAACTTGAGGCAGATA :  390
BnLPAT5-UK : TGGGAACATGAAGTATGTGCTTAAGAGCAGCTTGATGAAGTTACCTCTGTTTGGTTGGGCGTTTCACCTCTTTGAGTTTATCCCCGTTGAGAGGAAATGGGAAGTCGATGAAGCGAACTTGAGGCAGATA :  390
             TGGGAA ATGAAGTATGT CTTAAGAGCAG  TGATGAAgtT CCTCTGTT GGTTGGGCGTTTCACCTCTTTGAGTTTATcCCCGTTGAGcGGAaATGG AAGT GATGAAGC AACTTGAGGCAGAT

                  400         *       420         *       440         *       460         *       480         *       500         *       520
BnLPAT5-A5 : GTTTCCAGTTTTAAGGATCCTCGAGATGGTTTATGGCTTGGTCTTTTTCCCGAGGGCACAGATTACAC-----------GCGAAAGGAGCAAGAAGTTTGCAGCTGAGAATGGCCTTCCAGAACTGAACA :  509
BnLPAT5-C5 : GTTTCCAGTTTTAAGGATCCTCGAGATGGTTTATGGCTTGGTCTTTTCCCCGAGGGCACAGATTACACAGAGGCGAAACGCGAAAGGAGCAAGAAGTTTGCAGCTGAAAATGGTTTACCCATACTGAACA :  520
BnLPAT5-C1 : GTTTCAAGTTTTAAGGATCCTAGAGACGCTTTATGGCTTGCTCTTTTCCCTGAAGGCACAGATTACAC-----------GCGAAAGGAGCAAGAAGTTTGCAGCCGAACATGGCTTACCAGTACTGAGCA :  509
BnLPAT5-UK : GTTTCAAGTTTTAAGGATCCTAGAGATGCTTTATGGCTTGCTCTTTTCCCCGAAGGCACAGATTACAC-----------GCGAAAGGAGCAAGAAGTTTGCAGCTGAACATGGCTTACCAGTACTGAACA :  509
             GTTTC AGTTTTAAGGATCCT GAGAtG TTTATGGCTTG TCTTTTcCCcGA GGCACAGATTACAC           GCGAAAGGAGCAAGAAGTTTGCAGCtGAa ATGGcTTaCCagtACTGAaCA

                      *       540         *       560         *       580         *       600         *       620         *       640         *
BnLPAT5-A5 : ATGTGTTGCTTCCCAAGACAAAAGGTTT-GTCTCCTGCTTGGAAGAATTGGATTCCTCACTTGATGCAGTTTATGATGTGACCATCGGTTACAAAACCCGTTGTCCGTCTTTCTTAGACAATGTCTATGG :  638
BnLPAT5-C5 : ACGTGTTGCTTCCCAAGACAAAAGGTTTCGTCTCCTGCTTGGAAGAACTGGGTTGCTCACTTGATGCAGTTTATGATGTGACCATCGGTTACAAAACCCGTTGTCCGTCTTTCTTAGACAATGTCTATGG :  650
BnLPAT5-C1 : ACGTGCTGCTTCCAAGGACTAAAGGTTTCGTCTCTTGCTTGCAAGAACTGAGCTCCTCACTTGATGCAGTTTATGACGTGACCATCGGCTATAAAACCCGTTGTCCGTCTTTCTTAGACAACGTCTATGG :  639
BnLPAT5-UK : ACGTCCTGCTTCCAAGGACTAAAGGTTTCGTCTCTTGCTTGCAAGAACTGAGTTCCTCACTTGATGCAGTTTATGACGTGACCATCGGTTATAAAACCCGTTGTCCGTCTTTCTTAGACAACGTCTACGG :  639
             AcGTg TGCTTCC A GAC AAAGGTTTcGTCTC TGCTTG AAGAAcTG gtTcCTCACTTGATGCAGTTTATGA GTGACCATCGGtTA AAAACCCGTTGTCCGTCTTTCTTAGACAA GTCTAtGG

                  660         *       680         *       700         *       720         *       740         *       760         *       780
BnLPAT5-A5 : TACTTGAACCATCAGAAGTTCACATCCACATCCGTCGAATCAGCCAAAACCAAATCCCAAATGAAGAAAAGGAAATCAATGCTTGGTTAATGAACACATTCCAGATCAAAGACCAGCTGCTCAGTGAGTTC :  768
BnLPAT5-C5 : TACTTGAACCATCAGAAGTTCACATCCACATCCGTCGAATCAGCCAAAACCAAATCCCAAATGAAGAAAAGGAAATCAGTGCTTGGTTAATGAACACATTCCAGATCAAAGACCAGCTGCTCAGTGAGTTC :  780
BnLPAT5-C1 : TATCGAACCATCAGAAGTTCACATCCACATCCGTCGAATCAACCAAAGCAAAATCCCAAATCAAGAAAAGGAGATCAATGATTGGTTAATGAACGCGTTTCAGCTCAAAGACCAGCTTCTCAGTGACTTC :  769
BnLPAT5-UK : TATTGAACCATCAGAAGTTCACATCCGAATCCGTCGAATCGACCAAAGCCAAATCCCAAATCAAGAAAAGGAGATCAATGATTGGTTAATGAACGCGTTCCAGCTCAAAGACCAGCTTCTCAGTGACTTC :  769
             TA tGAACCATCAGAAGTTCACATCCacATCCGTCGAATCa CCAAA CcAAATCCCAAAT AAGAAAAGGA ATCAaTG TTGGTTAATGAAC C TTcCAG TCAAAGACCAGCT CTCAGTGA TTC

                      *       800         *       820         *       840         *       860         *       880         *       900         *
BnLPAT5-A5 : TACTCTCGTGGCCATTTCCCTAACGAAGGAACAGAAAAAGAGTTCAGCACAATAAAGCAACTCATAAACTGCTTGGCGGTGATTGTGTTCACGATCATCTGCACGCATCTCACCTTCTTCTCGTCGATGA :  898
BnLPAT5-C5 : TACTCTCGTGGCCATTTCCCTAACGAAGGAACAGAAAAAGAGTTCAGCACAATAAAGCAACTGATAAACTGCTTGGCGGTGATTGTGTTCACGATCATCTGTACGCATCTCACCTTCTTCTCGTCGATGA :  910
BnLPAT5-C1 : TACTCCAGTGGCCATTTCCCTAACGAAGGAACAGAAAAAGAGTTCAACACACTGAAGCACCTCATAAACTGCTTGGCAGTGATTTTCTTCACGACCATCTGCACGTATCTCACCTTCTTTTCGTCAATGA :  899
BnLPAT5-UK : TACTCCAGTGGCCATTTCCCTAACGAAGGAACAGAAAAAGAGTTCAACACACTGAAGCACCTCATAAACTGCTTGGCAGTGATTGTCTTCACGGTCATCTGCGCGTATCTCACCTTCTTTTCGTCGATGG :  899
             TACTC  GTGGCCATTTCCCTAACGAAGGAACAGAAAAAGAGTTCA CACA T AAGCA CTcATAAACTGCTTGGC GTGATTgT TTCACGatCATCTGcaCG ATCTCACCTTCTT TCGTCgATGa

                  920         *       940         *       960         *       980         *      1000         *      1020         *      1040
BnLPAT5-A5 : TCTGGTTCAAGATATATGTCTCTTTGGTCTGTGCCTACTTGACCTATGCTACTCATTTCAATCTTCGTCCTGCGCCTCTTGTTGAGACTGCAAAGAAAAGCTTT------AGTAAAAAATTGAACTTTTA : 1022
BnLPAT5-C5 : TCTGGTTCAAGATCTATGTCTCTTTGGTCTGTGCCTACTTGACCTGTGCTACTCATTTCAATCTTCGTCCTGCGCCACTTGTTGAGACTGCAAAGAAAAGCTTTCAAATTAGTAAAAAAATGAACTTTTA : 1040
BnLPAT5-C1 : TCTGGTTCAAGATCTCTGTCTCTTTGGTCTGTGCCTACTTGGCCTCCGCTACACATTTCAATCTTCGTTCTGTTCCACTTGTTGGGACTGCAAA-AAAAGCCTTCAAAT--------------------- : 1007
BnLPAT5-UK : TCTGGTTCAAGATCTATGTCTCTTTGGTCTGTGCCTACTTGGCCTCCGCTACACATTTCAATCTTCGTTCTGTTCCACTTGTTGAGACTGCAAA-AAAGGCCTTCAAATGATTAA--------------- : 1013
             TCTGGTTCAAGATcTaTGTCTCTTTGGTCTGTGCCTACTTG CCT  GCTAC CATTTCAATCTTCGT CTG  CCaCTTGTTGaGACTGCAAA AAAaGC TTcaaat a taa
```

BnLPAT5-A5	:	A	:	1023
BnLPAT5-C5	:	A	:	1041
BnLPAT5-C1	:	-	:	-
BnLPAT5-UK	:	-	:	-
