# Supplementary figures and images for: Effective editing for lysophosphatidic acid acyltransferase 2/5 in allotetraploid rapeseed (Brassica napus L.) using CRISPR-Cas9 system
Source: Biotechnol Biofuels. 2019 Sep 20;12:225. doi: 10.1186/s13068-019-1567-8 (PMC6753616; doi:10.1186/s13068-019-1567-8)

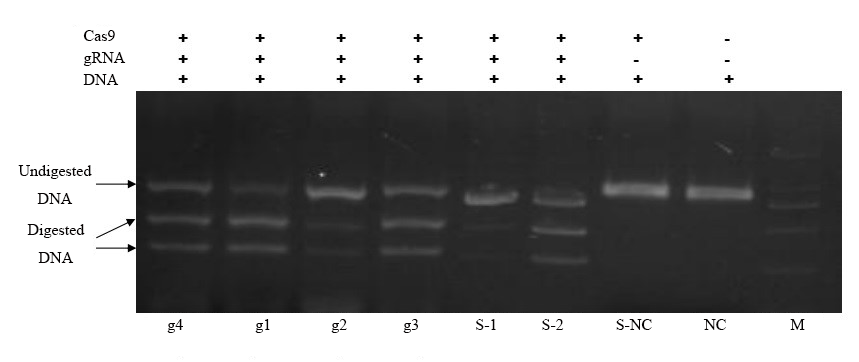

Supplement: Supplementary file 4 — Additional file 4. In vitro assay of three guide RNAs mediated Cas9 activity. The arrow represents the digested fragments. M, 1000 bp DNA marker. S-1 and S-2 mean standard gRNA, whose SSA activity is 30% and 100%, respectively. [file 13068_2019_1567_MOESM4_ESM.tif]

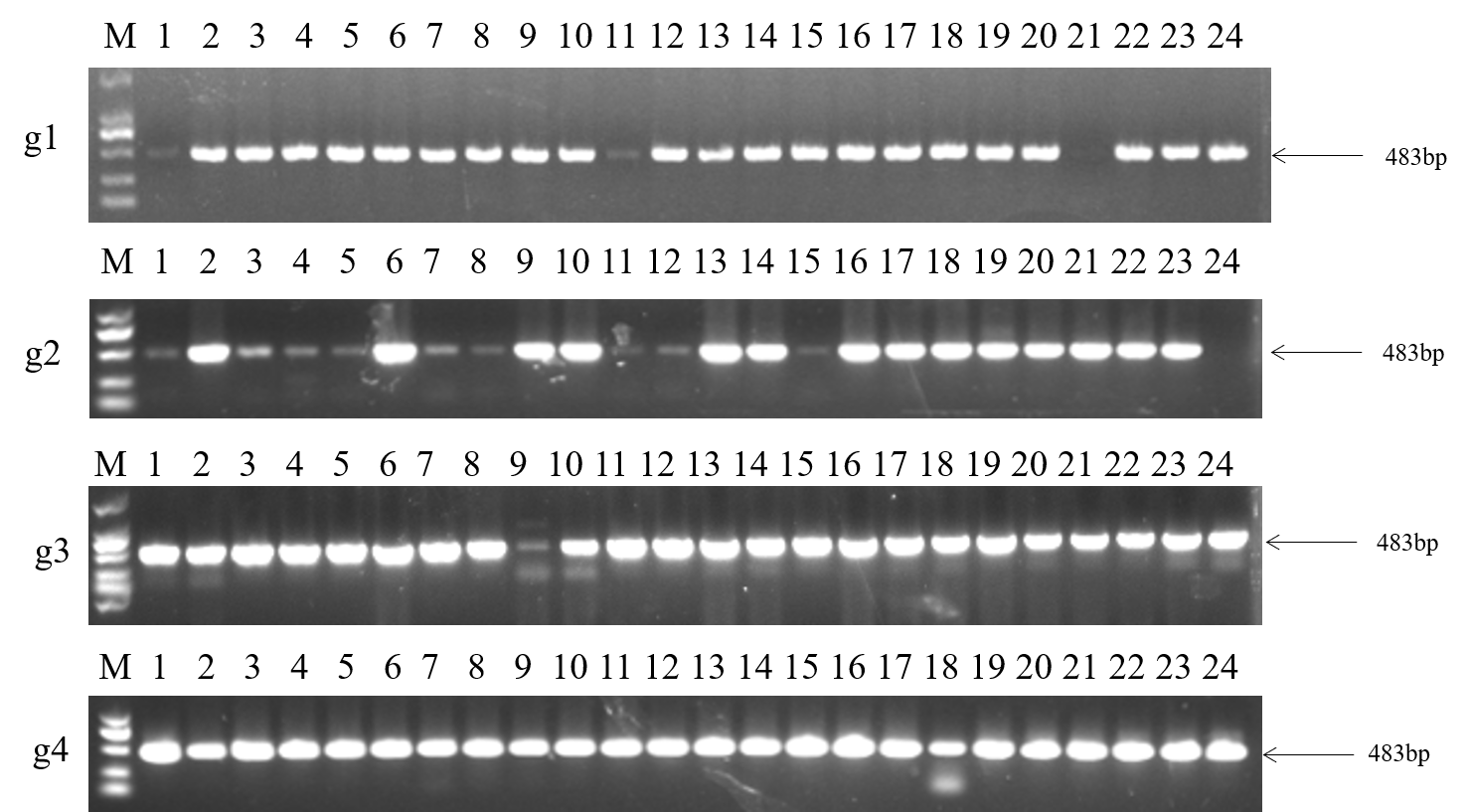

Supplement: Supplementary file 5 — Additional file 5. PCR detection gene insertion in the T0 generation. Cropped gel image showing the PCR products of gRNA-sac in different progeny from g1, g2, g3 and g4 lines. Marker, DL 2000 bp. −: gDNA from WT was used as a negative control. [file 13068_2019_1567_MOESM5_ESM.tif]

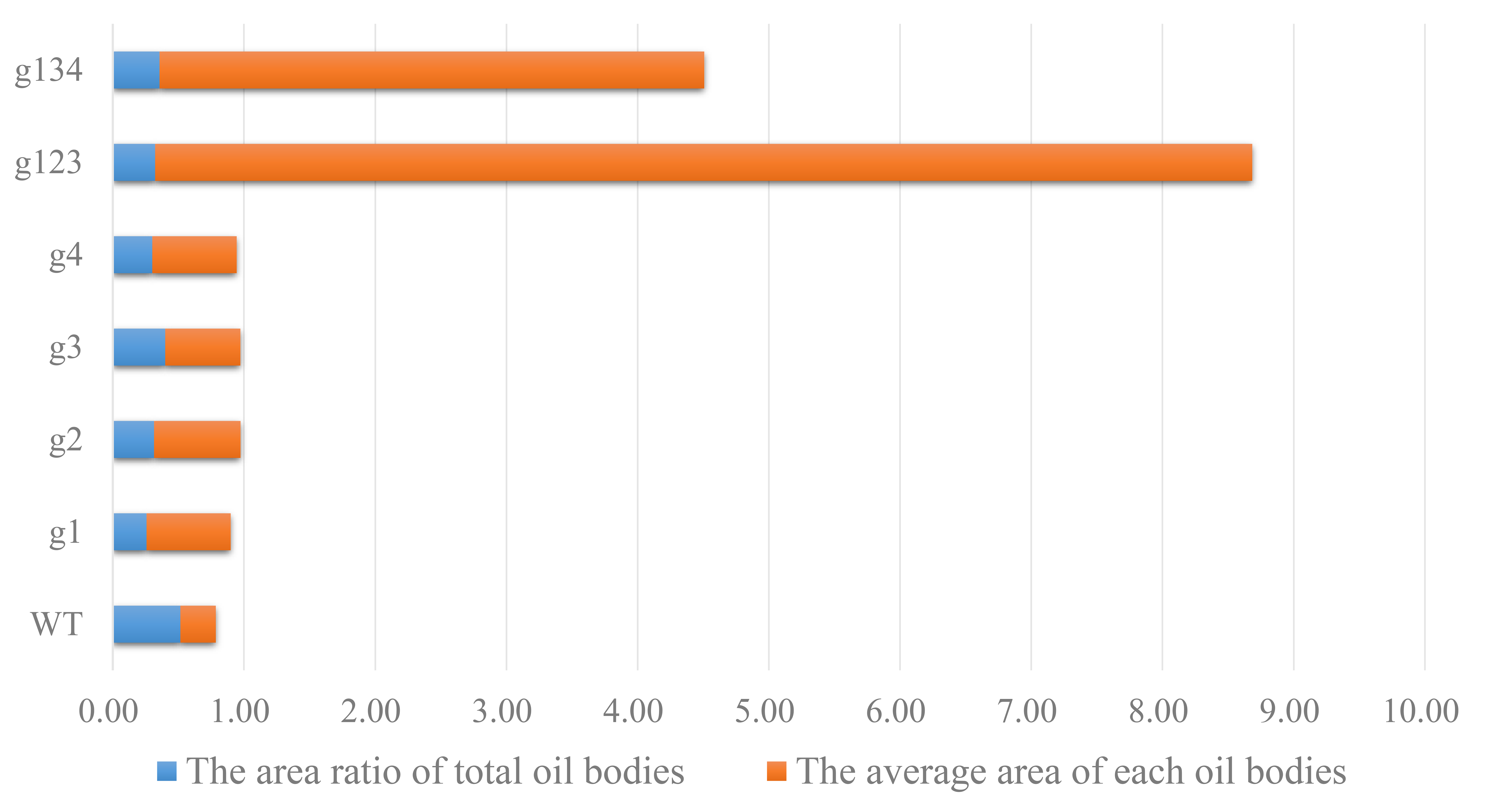

Supplement: Supplementary file 9 — Additional file 9. The area ratio of total oil bodies and the average size of each oil body. [file 13068_2019_1567_MOESM9_ESM.tif]
